# mKmer: an unbiased *K*-mer embedding of microbiomic single-microbe RNA sequencing data

**DOI:** 10.1093/bib/bbaf227

**Published:** 2025-05-23

**Authors:** Fangyu Mo, Qinghong Qian, Xiaolin Lu, Dihuai Zheng, Wenjie Cai, Jie Yao, Hongyu Chen, Yujie Huang, Xiang Zhang, Sanling Wu, Yifei Shen, Yinqi Bai, Yongcheng Wang, Weiqin Jiang, Longjiang Fan

**Affiliations:** Hainan Institute, Zhejiang University, Zhenzhou Road, Yazhou Bay Science and Technology City, Yazhou District, Sanya 572025, Hainan Province, China; Institute of Crop Science, Zhejiang University, 866 Yuhangtang Road, Xihu District, Hangzhou 310058, Zhejiang Province, China; Institute of Crop Science, Zhejiang University, 866 Yuhangtang Road, Xihu District, Hangzhou 310058, Zhejiang Province, China; Institute of Bioinformatics and James D. Watson Institute of Genome Sciences, Zhejiang University, 866 Yuhangtang Road, Xihu District, Hangzhou 310058, Zhejiang Province, China; Institute of Crop Science, Zhejiang University, 866 Yuhangtang Road, Xihu District, Hangzhou 310058, Zhejiang Province, China; Liangzhu Laboratory (Zhejiang Provincial Laboratory for Systems Medicine and Precision Diagnosis), Zhejiang University, 1369 Wenyi West Road, Yuhang District, Hangzhou 311121, Zhejiang Province, China; Institute of Bioinformatics and James D. Watson Institute of Genome Sciences, Zhejiang University, 866 Yuhangtang Road, Xihu District, Hangzhou 310058, Zhejiang Province, China; Institute of Crop Science, Zhejiang University, 866 Yuhangtang Road, Xihu District, Hangzhou 310058, Zhejiang Province, China; Institute of Crop Science, Zhejiang University, 866 Yuhangtang Road, Xihu District, Hangzhou 310058, Zhejiang Province, China; Department of Colorectal Surgery, The First Affiliated Hospital, Zhejiang University School of Medicine, 79 Qingchun Road, Shangcheng District, Hangzhou 310003, Zhejiang Province, China; Analysis Center of Agrobiology and Environmental Sciences, Zhejiang University, 866 Yuhangtang Road, Xihu District, Hangzhou 310058, Zhejiang Province, China; Department of Laboratory Medicine, The First Affiliated Hospital, Zhejiang University School of Medicine, 79 Qingchun Road, Shangcheng District, Hangzhou 310003, Zhejiang Province, China; BGI-Sanya, Zhenzhou Road, Yazhou Bay Science and Technology City, Yazhou District, Sanya 572025, Hainan Province, China; Liangzhu Laboratory, Zhejiang University, 1369 Wenyi West Road, Yuhang District, Hangzhou 311113, Zhejiang Province, China; Department of Colorectal Surgery, The First Affiliated Hospital, Zhejiang University School of Medicine, 79 Qingchun Road, Shangcheng District, Hangzhou 310003, Zhejiang Province, China; Hainan Institute, Zhejiang University, Zhenzhou Road, Yazhou Bay Science and Technology City, Yazhou District, Sanya 572025, Hainan Province, China; Institute of Crop Science, Zhejiang University, 866 Yuhangtang Road, Xihu District, Hangzhou 310058, Zhejiang Province, China; Institute of Bioinformatics and James D. Watson Institute of Genome Sciences, Zhejiang University, 866 Yuhangtang Road, Xihu District, Hangzhou 310058, Zhejiang Province, China

**Keywords:** *K*-mer, smRNA-seq, HCK, reference genome-free, *K*-motif

## Abstract

The advanced single-microbe RNA sequencing (smRNA-seq) technique addresses the pressing need to understand the complexity and diversity of microbial communities, as well as the distinct microbial states defined by different gene expression profiles. Current analyses of smRNA-seq data heavily rely on the integrity of reference genomes within the queried microbiota. However, establishing a comprehensive collection of microbial reference genomes or gene sets remains a significant challenge for most real-world microbial ecosystems. Here, we developed an unbiased embedding algorithm utilizing *K*-mer signatures, named mKmer, which bypasses gene or genome alignment to enable species identification for individual microbes and downstream functional enrichment analysis. By substituting gene features in the canonical cell-by-gene matrix with highly conserved *K*-mers, we demonstrate that mKmer outperforms gene-based methods in clustering and motif inference tasks using benchmark datasets from crop soil and human gut microbiomes. Our method provides a reference genome-free analytical framework for advancing smRNA-seq studies.

## Introduction

Recently, we developed a high-throughput single-microbe RNA sequencing (smRNA-seq) technique for microbiome samples [1], which can generate RNAs from over 5000 single microbes of a microbial community. This technique can effectively solve the problem of significant cell heterogeneity among bacterial populations and thus achieve complete functional characterization of host-related microorganisms. However, there is a key challenge in the smRNA-seq data analysis: constructing a high-quality gene expression matrix for downstream analysis [2]. Typically, the construction of a gene expression matrix requires a reference genome [3]. While the human reference genome is largely complete and accurate, this is not the case for other organisms. For example, in the case of microorganisms, the quality of various microbial genomes has been greatly improved with the help of metagenome-assembled genomes (MAGs) [4] and single-amplified genomes (SAGs) [5]. However, for some microorganisms, the quality of their reference genomes and annotated gene sets still remains uncertain [6]. Additionally, the rapid evolutionary rate of microorganisms can result in many true variant reads failing to be aligned to the reference genome. Therefore, relying solely on a single reference genome may not be the optimal solution for all samples. These issues are especially pronounced when sequencing data encompasses multiple unclassified species (e.g. microbiome data). Any attempt to predefine which species should be included in the reference genome(s) inevitably introduces bias.

*K*-mer refers to short sequences of a specific length (*K*) and any genomic or RNA sequences are composed of different *K*-mers. *K*-mers have been widely used in bioinformatics analysis, including genome survey and assembly, and also single-cell omics data [e.g. [7–9]]. Theoretically, RNAs from a microbiome sample can be characterized by a specific length of short sequence (i.e. *K*-mers). In this study, we developed a new frame of smRNA-seq analysis (named mKmer) based on high-frequency conserved *K*-mer (HCK) rather than a gene expression matrix. Benchmark tests on seven datasets from soil and gut demonstrate that mKmer significantly improved species identification compared to the cell-by-gene matrix. To demonstrate applications of our method, we used clinical smRNA-seq data from the gut microbiome of colorectal cancer patients before and after treatment.

## Materials and methods

### Single-microbe RNA sequencing data collection and generation

A total of seven smRNA-seq datasets, four from healthy donors by our previous study [1] (PRJCA017256), two from a patient, and one from soybean soil generated by this study, were used for performance and benchmarking of mKmer. These two fecal samples were collected from the same patient with colorectal cancer, both before and after immunotherapy. The study protocol was approved by the Ethics Committee of the First Affiliated Hospital, Zhejiang University School of Medicine, China (2021IIT A0239). The protocols for sample treatment for high-throughput smRNA-seq followed our previous study [1] and the smRNA-seq data were generated by M20 Genomics platform.

### Quality control of raw data

UMI-tools (v1.1.4) [10] were used to process the unique molecular identifiers (UMIs) of our smRNA-seq data. We utilized the *umi-tools whitelist* for quality control of the raw data to estimate the number of cells accurately. Given that the raw data file R1 is ~1 GB, we specified an expected cell number before determining the actual count. Therefore, the *--expect-cells* parameter was set to 10 000. The raw data had a barcode length of 20 bp and a UMI length of 8 bp. To obtain the whitelist, the *--bc-pattern* was set to *CCCCCCCCCCCCCCCCCCCC NNNNNNNN*, and the *--set-cell-number* parameter was set to 7000, which corresponded to the cell number at the inflection point in the barcode rank plot. We then used the *umi-tools extract* to filter the raw data files R1 and R2 based on the obtained whitelist, resulting in cleaned raw data.

During the PCR process of smRNA-seq, some molecules may have been disproportionately amplified due to sequence characteristics (e.g. Guanine and Cytosine (GC) content) or random factors, resulting in multiple reads with the same barcode and UMI. To address this, we employed the *RemoveDuplicates* within UMI-tools to retain only the highest-quality read, as determined by the Phred quality scoring system, among those with the same barcode and UMI. The sequencing data distinguish each read in the form of 20bp_8bp, so the *RemoveDuplicates* defaults to the last 29 characters of the sequence information line in the FASTQ (FASTQ format: A text-based standard for storing nucleotide sequences and their quality scores, containing four lines per record: (1) @-prefixed identifier, (2) nucleotide sequence, (3) +-prefixed separator, (4) ASCII-encoded quality scores) file as a unique identifier.


$$ {Q}_{avg}=\frac{1}{\left|Q\right|}\sum_{i=1}^{\left|Q\right|}\left(\mathrm{ord}\left({Q}_i\right)-33\right) $$

$$ \mathrm{if}\ ID\notin \mathrm{seqs}\ \mathrm{or}\ {Q}_{avg}>\mathrm{seq}\left[ ID\right]\left[\mathrm{quality}\right]\ \mathrm{then} $$

$$\mathrm{seqs}\left[ ID\right]=\left\{\mathrm{seq}:S,\mathrm{quality}:{Q}_{avg}\right\}$$

Let *S* denote a sequence in the FASTQ file (consisting of four lines); *ID*(*S*) denotes the unique identifier of the sequence *S* (the last 29 characters of the first line, i.e. barcode + UMI); *Q*(*S*) denotes the quality string line of the sequence *S* (the fourth line of the four lines).

### *K*-mer scanning and counting

Jellyfish (v2.2.10) [11] was used for fast, memory-efficient counting of *K*-mers in DNA sequences. In our experiment, the *--m* parameter of the *jellyfish count* was set to the default value of 10, to determine the *K*-mer length (*K*). This *K* corresponds to the smallest *K* where the peak in *K*-mer frequency distribution occurs at *x* = 1. The *--s* parameter was set to the default value of 10 M. To ensure detection of all high-frequency *K*-mers, the *--h* parameter was set to 100 000 000 based on experimental testing.

To confirm that the selected *K* value was reasonable, we observed the distribution of peaks with different *K* values by drawing KmerFrequency plots. The *--put* of the *KmerFrequency* was three histo files with different *K* values specified, and the *--out* argument specified the path to the output KmerFrequency plot file. The histo file generated with the selected *K* was used as the input for the *KmerRank* to create a *K*-mer rank plot, where the *x* value at the inflection point indicates the number of HCKs. We first transformed *K*-mer cumulative frequency and count into logarithmic scales and then calculated first-order derivatives within the central 80% data range. The knee point was identified based on the position with maximum absolute derivative. The *jellyfish dump* was then used to convert the *jf* file into a readable format for extracting the top-counts *K*-mers.


$$\mathrm{HCK} = \mathrm{select\_top}_N\left(\mathrm{sort}\big[\mathrm{count}(Kmers, C), \mathrm{desc}\big]\right)$$

$$M_{ji} = \mathrm{count}({HCK}_i, {C}_j)$$

In the frequency matrix *M*, a specific element *M_ji_* represents the occurrence count of the *i*-th HCK in the *j*-th cell among the selected HCKs.

### Generation of cell-by-high-frequency conserved *K*-mer matrix

After detecting each *K*-mer, they were sorted by detection depth in descending order. HCKs were then selected based on this sorted list. Using the generated HCK list, *K*-mers were read sequentially from the cleaned R2 reads.


$$T_k = \sum_{i=1}^{N} C_{i,k}$$

*C_i,k_* represents the count of *K*-mers in the *i*-th sequence of the FASTQ file.


$$A_k = \sum_{i=1}^{N} \mathbb{I}(C_{i,k} > 0)$$

*A_k_* represents the number of times the *K*-mer appears in the FASTA file, where *A_k_* is 1 if the *K*-mer appears in the *i*-th sequence and 0 otherwise.


$$ \text{cell}\_\text{seq}\_\text{vector} [c][i] = \sum_{j=1}^{L-K+1} \mathbb{I}(s[j:j+K-1] \in \mathcal{K}_i)$$

Then, a sequence vector for each cell is generated. *L* is the length of the sequence, *K* is the length of the *K*-mer, and *Kmers* is the set of selected *K*-mers.

Finally, sort in descending order according to Tk and select the top M *k*-mers to generate the cell-by-HCK matrix. To minimize memory usage during execution, the program generated a cell-by-HCK matrix for every 1000 cell reads and then merged these matrices. For ease of use, we integrated the entire process of cell-by-HCK matrix into a single command named *KmerCell*. The *--kmercount* argument required the *kmer_counts_dumps.fa* file output from jellyfish dump (the file suffix must be *_counts_dumps.fa*); *--fastq* required the clean R2 FASTQ file; *--topkmer* specified the number of HCKs; and *--k* specified the selected *K*.

### Identification of microbial species

We employed a *K*-mer-based root-to-leaf classification strategy for microbial species annotation, which is integrated in our software under the name *smAnnotation*. We first applied Kraken 2 (v 2.0.7-beta) [12], a *K*-mer-based read classification method, on every read in each barcode based on the standard refseq of Kraken 2(Refseq archaea, bacteria, viral, plasmid, human1, https://benlangmead.github.io/aws-indexes/k2). After all the reads were assigned to each node of different taxonomic levels (e.g. order, family, genus, species), we calculated the sum of reads in each node from the leaf to the root. Then, we performed the taxonomic classification from the root to the leaf taxonomic levels. In the root taxonomic level, we ranked all the nodes from the highest to the lowest based on the number of reads of the nodes, and selected the node with the most reads as a potential annotation candidate. Based on the annotation results, we then performed the same annotation process in the next lower taxonomic level, until the leaf nodes (species level). Then, Bracken (v 2.5) [13] was used to count the *fraction_total_reads* of the species classified into each cell, and the species with the largest value was selected as the final annotation result. For Kraken 2, the comparison database was the National Center for Biotechnology Information (NCBI) standard database by default (Archive size: 60 GB) and the resolution parameter *--r* was set to 100. For *smAnnotation*, clean R2 as the specified file for *--input*, and the output file named *smAnnotation.report* was placed in the current working directory by default.

### Visualization and clustering

To visualize the data, we further reduced the dimensionality of all filtered cells using Seurat (v4) [14] and used uniform manifold approximation and projection (UMAP) to project the cells into 2D space. The annotation results of Kraken 2 and Bracken were mapped to the Seurat object; only the annotation results with *fraction_total_reads* values >0.5 were retained, and strains with abundance <0.1% were filtered out. The steps include: (i) Using the LogNormalize method of the *NormalizeData* of Seurat to calculate the expression values of *K*-mers. The scale.factor argument is set to the default 10 000, nfeatures to 6000, and the *ScaleData* object to all genes; (ii) PCA was performed using the normalized expression value; among all the principal components, the top 30 principal components were used to do clustering and UMAP analysis; (iii) To find clusters, a weighted graph-based clustering method, Shared Nearest Neighbour (SNN), was selected, and the resolution is set to 0.5. Marker genes for each cluster were identified with the MAESTRO test with default parameters via the *FindAllMarkers* in Seurat, and the min.pct parameter was set to 0.25.

### Gene Ontology annotation by marker *K*-mers and motifs

Before performing functional enrichment analysis on sequencing data from different samples, we first need to filter out Ribosomal Ribonucleic Acid (rRNA) from the raw transcriptome data. In this study, we used SortMeRNA (v4.2.0) [15] for filtering, with the reference dataset including multiple species, such as bacteria, eukaryotes, archaea, and various sequencing databases, for 5S, 16S, 23S, and other rRNA types. After using the *FindAllMarkers*, we obtained a list of marker *K*-mers, which were used for functional enrichment analysis of clusters or species of interest. Marker *K*-mers are considered to be identified from highly conserved sequences, which are likely to represent individual motifs. The functional analysis of bacterial species based on their specific motifs is reliable. The MEME (Multiple Em for Motif Elicitation) suite (v5.0.5) [16] is a comprehensive resource for discovering and analyzing sequence motifs in DNA, RNA, and protein sequences. Memes [17] is an R package that provides a seamless R interface to a selection of popular MEME Suite tools. By analyzing the conserved sequences of each strain, we aimed to elucidate the specific functions of the strains. To obtain the specific functions of bacterial species, we designed two partitioning workflows to conduct Gene Ontology (GO) enrichment analysis on *K*-mer-contained motifs (*K*-motifs).

#### Nucleotide motif analysis (KmerGOn)

The first analysis workflow involved converting each marker *K*-mer into a motif file in MEME format. These motifs were then compared against motifs in the microbial nucleotide motif database using the *tomtom* [18] integrated into the MEME suite. This step identified the best-matching known motifs. Subsequently, *ama* and *gomo* [19] in the MEME suite were used to compare the identified known motifs against the *Escherichia coli* database, obtaining GO functions for each motif. Finally, GO functional enrichment and visualization were performed on the clusters or species of interest.

#### Protein motif analysis (KmerGOp)

The second analysis workflow utilized SeqKit (v2.8.2) [20, 21] to translate each marker *K*-mer into amino acids using six reading frames (since *K* = 12, only sequences with 4 AA were retained). These motifs were then compared against motifs in the all-species motif database using the *tomtom* integrated into the MEME suite. Each protein motif was further analyzed using InterProScan (v5.47-82.0) [22] to search domain databases (e.g. Pfam, PROSITE, PRINTS) and obtain GO IDs. Finally, used *select* of the AnnotationDbi (v1.64.1) [23] package to match the corresponding term and GO ID from the GO.db (v3.18.0) [24] database.

For both workflows, the output list of marker *K*-mers from the *FindAllMarkers* served as the input file. The --*cluster* was set to the target cluster, and the --*out* specified the path to the output file. This ensured a systematic approach to uncovering the functional roles of conserved sequences within bacterial species.

## Results

### Overview of mKmer method

**Figure 1:**
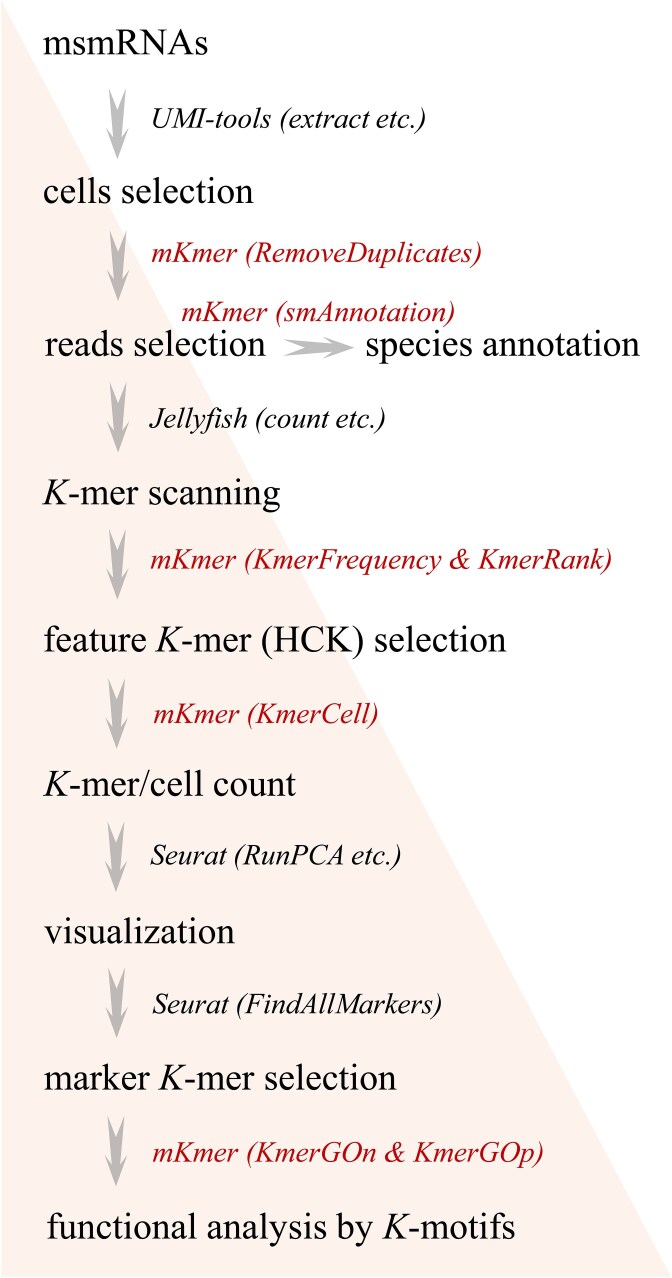
Overview of the mKmer method. There are seven modules in mKmer for msmRNA-seq data analysis, including read selection, species annotation, selection of K and HCKs, cell-by-HCK matrix construction, and functional analysis. Traditional analysis software, such as UMI-tools, Jellyfish, and Seurat, are also employed in the mKmer pipeline.

**Figure 2:**
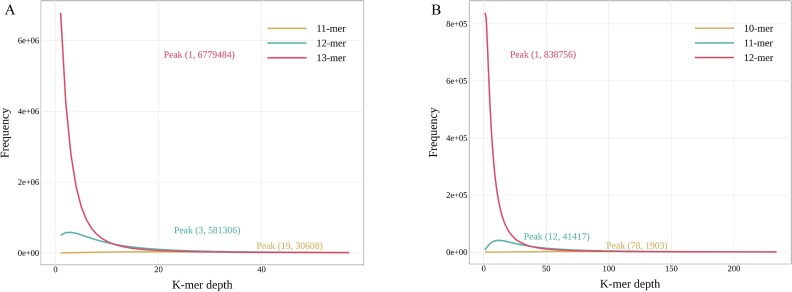
Frequency of *K*-mer depths by different *K* sizes for msmRNA-seq datasets from soybean soil (A) and human gut (B) samples. The *X*-axis represents the number of times *K*-mers are detected, and the *Y*-axis indicates the number of such *K*-mers.

mKmer is a tool for smRNA-seq data analysis by constructing a cell-by-HCK matrix to achieve efficient cell classification, species annotation, and functional analysis. The tool is currently divided into seven analysis modules, including *KmerRank* and *KmerCell*, aiming to provide personalized services for analysts (Fig. 1). mKmer extracts biological information from raw sequencing data by testing different *K*-mer lengths to determine the optimal *K* (Fig. 2). This optimal *K* can identify key conserved sequences while effectively distinguishing noise from noncritical gene sequences. In our analysis framework, we successfully identified species and performed functional analysis for soybean soil samples and human fecal samples by selecting these HCKs before the inflection point. This demonstrates that HCKs already densely contain a large amount of hierarchical information in microbial taxonomy, enabling precise and efficient classification at various levels, including domain, phylum, class, order, family, genus, and species. On the other hand, the low-frequency *K*-mers contain sparse taxonomic information, and discarding them does not affect the classification results. Therefore, HCKs may be translated into amino acid sequences (i.e. protein motifs) accordingly. Additionally, HCKs address issues of computational time and high matrix dimensionality caused by the excessive variety of *K*-mers, which significantly enhanced mKmer’s performance and practicality. Because it does not require any reference genome, the reproducibility of sequencing reads reached 100%.

#### *K*-mer scanning and rank plot

**Figure 3:**
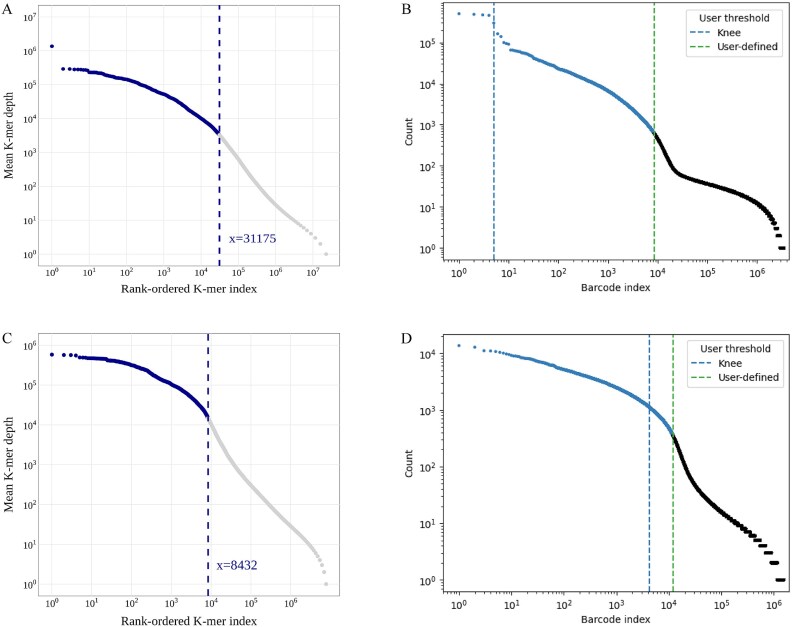
*K*-mer rank plot for HCKs (the *X*-axis represents the position (*x*) of *K*-mers sorted in descending order by detection count, and the *Y*-axis indicates the average detection count of the top *x K*-mers) and barcode rank plot (the *Y*-axis coordinates represent UMI count) calling of the smRNA-seq data. (A) *K*-mer rank plot of a soybean soil smRNA-seq (*K* = 13). (B) Barcode rank plot of the soybean soil smRNA-seq. (C) *K*-mer rank plot of a human gut smRNA-seq (*K* = 12). (D) Barcode rank plot of the human gut smRNA-seq.

For a raw single-microbe RNA (smRNA) dataset, cells were selected as usual (such as with UMI-tools), and reads from the selected cells were filtered to remove duplicates before the downstream *K*-mers scanning (Fig. 1). We tested frequency distributions of different *K*-mer sizes for the seven smRNA datasets and found a change of distribution curves happening between 12-mer and 13-mer in all samples (Fig. 2, for the other five samples, see Supplementary Fig. S1). Due to the high-throughput smRNA-seq technique using random primers to capture RNA of individual cells, the combined amplification and release yield an average RNA sequencing depth of 1× coverage. Therefore, low-frequency *K*-mers with a frequency of 1, where the *K* value is at its peak, are considered optimal. The 12-mer was therefore used as the default size for *K*-mers scanning. We further ranked all 12-mers or 13-mers scanned from the smRNA data by count per cell (Fig. 3, for the other five samples, see Supplementary Fig. S2). The *K*-mer rank plot (from highest to lowest *K*-mer depth) is an interactive plot that shows all *K*-mers detected in a microbiome sample or a smRNA-seq dataset.

#### Identification of high-frequency conserved *K*-mers

**Figure 4:**
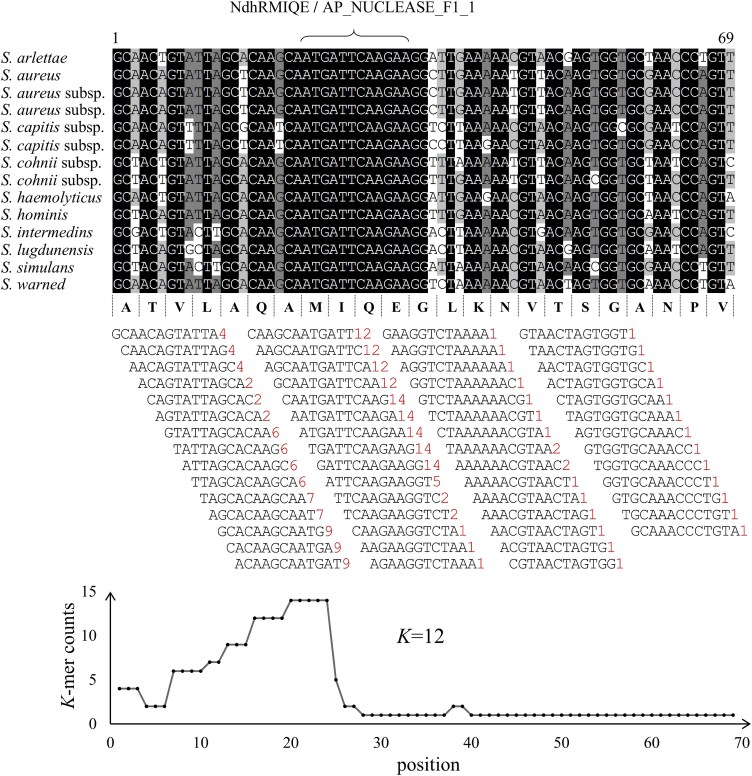
An example of HCKs. A conserved region coding for a motif of the bacterial gene HSP60 (top), and its counts of 12-mers obtained by scanning Staphylococcus, displayed as a line chart to show the distribution of HCKs (bottom). The motif’s names (nuclear/protein) from the MEME database are shown at the top. In the middle, the 12-mer composition of the conserved region (S. warned as reference) is shown. The numbers of each 12-mer are provided at the end of 12-mers.

The overall shape of the *K*-mer rank plot (Fig. 3A and C) is similar to the barcode rank plot (Fig. 3B and D). Typically, a “cliff-and-knee” shape can be observed in the *K*-mer rank plot of a microbiome sample. In this case, the steep cliff, followed by the plateaued knee, demonstrates that the *K*-mer calling algorithm was able to distinguish feature *K*-mers from others. HCKs mainly come from the evolutionarily conserved regions (e.g. motifs in protein domains and DNA-binding sites) of bacterial species in a microbiome sample. As an example, the region of the genus *Staphylococcus HSP60* gene encodes the conserved NdhRMIQE motif [25], and a high number of *K*-mers could be counted within this region (Fig. 4). The conserved *K*-mers exist in a wide variety of microbe species in a microbiome. From a microbial taxonomy perspective, the HCK corresponds to the lowest common ancestor (LCA) sequence at the taxonomic level. The use of *K*-mers for microbial classification has been demonstrated by many classical *K*-mer-based metagenomic taxonomy annotation software, such as Kraken [26], Centrifuge [27], and Kaiju [28]. The HCK was first discovered and successfully applied in the identification of taxa in single-cell data.

#### Identification of maker *K*-mers

**Figure 5:**
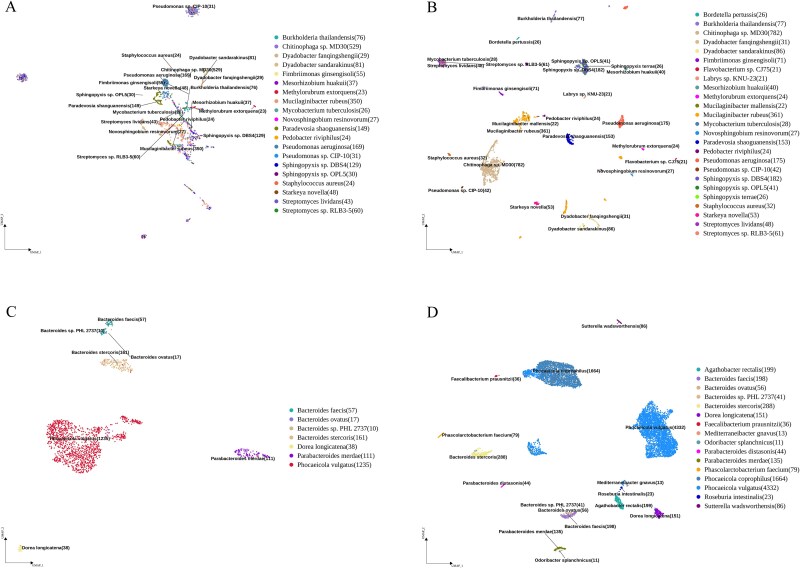
. Benchmark results of two samples by cell-by-gene matrix (A and C) and mKmer (B and D). UMAP clustering and species annotation of the msmRNA-seq data from the soybean soil (A) and human gut (B) samples. The numbers in parentheses are the number of cells of the strain.

Based on the cell-by-HCK matrix and routine cell clustering and dimension reduction (as shown in Fig. 1), a visualization result by the UMAP of an smRNA sample and species annotation can be obtained (examples shown in Fig. 5B and D, and the cell-by-gene matrix clustering results of this sample are shown in Fig. 5A and C). A good clustering of the same species/cells was observed in the UMAP plot. Further, marker *K*-mers can be identified among the different clusters (species or subspecies) using routine approaches (same as those for marker genes) such as the Seurat function (*FindAllMarkers*).

#### Function annotation by motifs

**Figure 6:**
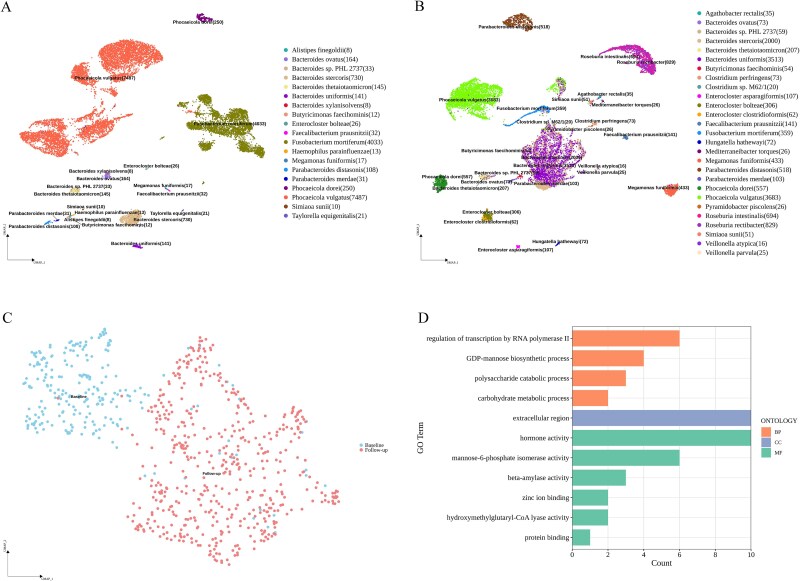
A case study by mKmer. (A, B) UMAP clustering and species annotation of the pretreatment (A) and posttreatment (B) gut msmRNA-seq data of a cancer patient using *K*-mers. (C) Integrated UMAP clustering of bacterial species *Phocaeicola. dorei* in the patient’s gut before (left) and after treatment (right) with mKmer. (D) Functional annotation of *P. dorei* in the gut of treated patients by KmerGOp.

The marker *K*-mer can be used for functional annotation based on their encoding motifs as mentioned above. Based on motif and domain databases (MEME and Pfam), the DNA motifs and protein motifs in domains can be identified for GO annotation by mKmer functions (*KmerGOn and KmerGOp*), respectively. At the protein level, the longest translated amino acids (AAs) (4-mer AA) for marker *K*-mers (12-mer nt) in a microbiome sample should have significant sequence similarity to the protein domain’s motifs in Pfam. The motif-contained *K*-mers are those highly conserved *K*-mers which transcript from the motif-contained regions of orthologous genes of different microbe species in a microbiome sample. Using the *K*-mer-contained motifs and their GO IDs identified, function analysis such as GO and pathway enrichment can be done as usual (Fig. 6D).

### Benchmark test with cell-by-gene matrix

To compare the performance of mKmer with the traditional gene matrix-based method, we generated a smRNA-seq dataset from soybean (*Glycine max*) soil and collected four publicly available smRNA-seq datasets from human guts. Firstly, when comparing the dimensionality reduction and clustering results using the traditional cell-by-gene matrix (two examples are shown in Fig. 5A and C; the other three examples of human gut are shown in Supplementary Fig. S4), regardless of whether the smRNA-seq data came from soil (Fig. 5A and B) or human gut (Fig. 5C and D), the cell-by-HCK matrix (Fig. 5B and D) was more distinct and accurate. In soil samples, numerous bacterial strains exhibited suboptimal separation in clustering analyses, with several taxonomically distinct species converging into central clusters. For instance, *Streptomyces sp. RLB3–5* (family *Streptomycetaceae*), *Burkholderia thailandensis* (family *Burkholderiaceae*), and *Methylorubrum extorquens* (family *Methylobacteriaceae*), which belong to divergent families, were erroneously grouped together in the cell-by-gene matrix clustering results (Fig. 5A). In contrast, the cell-by-HCK matrix effectively resolved these three species into three distinct clusters (Fig. 5B). A similar clustering result was also observed for *Pedobacter riviphilus*, *Mucilaginibacter rubeus* and *Sphingopyxis* sp. *DBS4*. The human gut microbiome dataset demonstrated ostensibly clear clustering patterns in the cell-by-gene matrix analysis, and the apparent resolution was achieved through implementation of excessively stringent filtering criteria that drastically reduced both microbial diversity and abundance. Although such rigorous filtering may enhance confidence in retained cellular data, it risks substantially misrepresenting the authentic complexity of microbial ecosystems by systematically excluding low-abundance taxa. We used the Davies–Bouldin Index [29] (DBI) and Silhouette Coefficient [30] (SC) to quantitatively evaluate clustering results for our seven smRNA-seq datasets. The results indicated that the clustering based on the cell-by-HCK matrix (mKmer) with a lower DBI or higher SC than the cell-by-gene matrix (STAR) (Supplementary Table S1), indicating better clustering efficacy or superior performance. Secondly, among our seven tested msmRNA-seq datasets, five exhibited a significantly higher number of species identified by the cell-by-HCK matrix compared to the cell-by-gene matrix, with five to nine microorganisms additionally identified per sample.

At the same time, the number of each species in both the soil and the gut also increased significantly. This is particularly evident for five species with low or moderate abundance (number < 300), including *Bordetella pertussis*, *Flavobacterium* sp. *CJ75*, and *Labrys* sp. *KNU-23* in the soybean soil sample. The additionally identified strains accounted for 5/21 of the original strains. In the human gut samples, nine types of microorganisms were additionally identified, including *Agathobacter rectalis*, *Faecalibacterium prausnitzii*, *Parabacteroides distasonis*, *Phascolarctobacterium faecium*, and *Sutterella wadsworthensis*. Therefore, mKmer shows significant advantages in species identification in both complex soil environments and gut environments. This advantage is particularly pronounced in datasets where the original results were not very good and the species diversity was relatively low. For systematic comparison, we mapped raw sequencing data to the reference genomes of the three most abundant microorganisms and five additionally identified species to determine their genome coverage. The total mapping rates for the additionally identified species ranged from 5.1% to 15.2% (Supplementary Table S2), confirming their authentic presence in our sequencing samples. Notably, *Sphingopyxis terrae* exhibited an exceptionally high unique mapping rate of 15.0%. Intriguingly, the microbe was undetectable in cell-by-gene matrix analyses. The failing was due to that the original soybean soil microbial reference genome lacked *S. terrae*. The mKmer approach resolves this limitation through its unbiased analytical framework, which enables species-agnostic characterization without prior knowledge of microbial community constituents. Moreover, we cumulatively identified 1164 unique *K*-mers across 23 microbial species. With the exception of *Mucilaginibacter mallensis*, unique *K*-mer signatures were successfully detected in all four additionally identified species (Supplementary File S1). Numerous studies have shown that the five bacterial species only identified by mKmer are commonly found in soil. *S. terrae* and *F.* sp. *CJ75* are capable of degrading complex organic compounds [31, 32]. *Mucilaginibacter mallensis* can produce mucilaginous polysaccharides, thereby improving soil structure and fertility [33]. *L.* sp. *KNU-23* is widely present in organic matter-rich soils [34]. Surprisingly, *B. pertussis*, primarily known as a human pathogen transmitted through the air, is not commonly found in soil environments, and its survival in soil has been seldom studied (10.1038/nrmicro886). The microbiome of the human gut is being studied more thoroughly. The species identified by mKmer, such as *A. rectalis*, *F. prausnitzii*, *Mediterraneibacter gnavus*, *Odoribacter splanchnicus*, *P. distasonis*, *P. faecium*, *Phocaeicola coprophilus*, *Roseburia intestinalis*, and *S. wadsworthensis*, are common human gut bacteria based on the literature [35–43]. Furthermore, genomic alignment of the corresponding reference species successfully confirmed the presence of these nine additionally identified microorganisms (Supplementary Table S3) and eight unique *K*-mers were exclusively identified in *Phocaeicola vulgatus* (Supplementary File S2).

To further validate the reliability of our results, we applied the mKmer functions *KmerGOn* and *KmerGOp* to in-depth explore the functions of the mKmer-identified species. The *B. pertussis* in soybean soil exhibits a unique ability to bind iron ions in soybean soil (Supplementary Fig. S3A). Iron is an essential micronutrient for plant growth, affecting soybean health and yield. Microorganisms can inhibit the growth of pathogens by competitively adsorbing iron in the soil, thereby reducing the occurrence of diseases. Compared to other microorganisms, acid phosphatase activity is significantly enriched in *B. pertussis* (Supplementary Fig. S3B), which can help decompose organic phosphorus compounds in the soil, releasing inorganic phosphorus that plants can absorb, thereby promoting phosphorus uptake by soybeans and enhancing crop growth. For human gut smRNA-seq data, the *A. rectalis* identified by mKmer revealed processes related to the metabolism of acids, including aconitate hydratase activity and the dicarboxylic acid metabolic process using *KmerGOn* (Supplementary Fig. S3C), consistent with findings by Abdugheni *et al*. [35]. In addition, several processes related to the biosynthesis of amines have been found by *KmerGOp*, such as 6-pyruvoyltetrahydropterin and tetrahydrobiopterin biosynthesis (Supplementary Fig. S3D). Taken together, mKmer doesn’t depend on reference genomes, and can unbiasedly and effectively parse the complex biological information in smRNA-seq data.

In the computational resource benchmark test, we allocated an equivalent number of computational threads (20 threads) to both mKmer and STAR. All smRNA-seq data processing and runtime operations were performed on a server equipped with a 64-core/128-thread Intel Xeon E7-4850 v4 processor (2.10GHz base frequency) and 1843 GB of memory. A detailed comparison of Central Processing Unit (CPU) time and maximum resident set size between mKmer and STAR (Table 1) revealed that mKmer exhibited significantly shorter runtimes than STAR when processing the smRNA-seq dataset, with performance improvements ranging from 19 to 98 times (an average improvement of ~48 times, a median of 44 times). The memory consumption during STAR operation showed a strong correlation with reference genome size. Since we cannot predetermine the microbial species in the samples, comprehensive reference genomes such as the Unified Human Gastrointestinal Genome (UHGG v2.0.2) [44] were employed. This approach consumes substantial computational resources (280.2 GB) during execution. Overall, mKmer demonstrates markedly superior computational efficiency.

**Table 1:** Computational resource comparison. From left to right are the sample names (encompassing all seven samples used in this study), input file sizes (R1 and R2), alignment software, user time consumed during computation, and maximum resident set size (max RSS).

**Sample**	**R1** **(GB)**	**R2** **(GB)**	**Software**	**User time** **(h)**	**Maximum resident set size** **(GB)**
Soybean soil	0.98	2.90	mKmer	0.30	7.40
			STAR	9.21	7.15
SAMC1266599	2.56	7.79	mKmer	0.35	8.20
			STAR	34.15	280.20
SAMC3766839	0.36	1.00	mKmer	0.27	2.20
			STAR	5.00	280.20
SAMC3766837	0.58	1.57	mKmer	0.34	5.08
			STAR	7.64	280.20
SAMC3766838	1.78	4.35	mKmer	0.38	2.18
			STAR	24.18	280.10
CRC patient (pretreatment)	2.64	6.81	mKmer	0.84	7.74
			STAR44	37.29	280.20
CRC patient (posttreatment)	3.47	8.86	mKmer	0.81	8.1
			STAR	51.86	280.20

Note. The identical maximum resident set size (max RSS) observed across the last five human microbiota samples is due to STAR’s automatic runtime memory allocation mechanism, which dynamically scales based on the size of the input reference genome.

### A case study using mKmer

To demonstrate the practical applications of mKmer, we collected fecal samples from a colorectal cancer patient before and after immunotherapy for single-microbe sequencing. Using mKmer to analyze the two smRNA-seq datasets, we identified 19 microbial species in the pretreatment fecal sample (Fig. 6A) and 27 species in the post-treatment sample (Fig. 6B, and the cell-by-gene matrix clustering results of these sample data are shown in Supplementary Fig. S5). The microbial richness increased by over one-third, which is conducive to the restoration of a healthy gut microbiota [45]. Through the analysis of these upregulated marker *K*-mers with *K*-mer-contained motifs, we found that hormonal regulatory activity and carbohydrate metabolic processes were enriched in the post-treatment sample (Supplementary Fig. S6). Further investigation into the shared species between the two samples, such as *Ph. dorei* (Fig. 6C), revealed that its populations in the pre- and post-treatment samples did not cluster together completely, indicating significant differences in gene expression. Consequently, we performed an in-depth analysis on functional changes in *Ph. dorei* between these two samples. GO enrichment results for marker *K*-mers in *Ph. dorei* (Fig. 6D) showed that functions related to polysaccharide metabolism and outer membrane-associated defense responses were significantly upregulated in the post-treatment sample. Studies have shown that short-chain fatty acids produced by polysaccharide metabolism suggest efficient therapy and good prognosis for colorectal cancer [46]. In addition, outer membrane binding and periplasmic space may promote biofilm formation, a process hypothesized to interact with host immune pathways [47, 48]. In a proteomics analysis, Kordahi *et al*. found that *Bacteroides fragilis*, via key functional proteins involved in lipopolysaccharide biosynthesis, induces pro-inflammatory cytokines by activating the TLR4/NF-κB pathway [49]. This pathway is directly correlated with the hormone activity (GO:0005179) and extracellular region (GO:0005576) identified in our results. Notably, *Ph. dorei* (formerly classified under *Bacteroides*) shares close phylogenetic proximity with *B. fragilis* and was reclassified as a distinct genus (*Phocaeicola*) [41]. Additionally, Cheng *et al*. employed an integrated analysis of fecal metagenomics and host proteomics to reveal that functional modules of gut microbiota in drug-resistant patients and found that purine/pyrimidine metabolism and glycerophospholipid metabolic pathways (e.g. PLA2G4A) were significantly enriched. These pathways are associated with carbohydrate metabolism (GO:0005975) and metalloenzyme activity (GO:0008270) [49]. Therefore, the innovative mKmer method can help researchers more comprehensively analyze the dynamic changes of intestinal microecology during immunotherapy and identify potentially beneficial microorganisms or their metabolites. These findings warrant validation in larger cohorts to explore therapeutic applications in colorectal cancer.

## Discussion

Our study presents mKmer, a novel reference genome-free approach for analyzing smRNA-seq data. The use of *K*-mer for species taxonomic identification has been demonstrated by many classical *K*-mer-based metagenomic taxonomy annotation software [26–28]. Although these tools classify species based on DNA, many conserved gene sequences remain highly consistent within species when DNA is transcripted into RNA. This implies that RNA sequences also contain species-specific conserved regions and are useful in *K*-mer analysis for species identification. For instance, rRNA and tRNA are widely used in taxonomic studies due to their significant conservation and variation among species [50, 51]. Messenger Ribonucleic Acid (mRNA), on the other hand, reflects gene expression, which varies significantly between species. By analyzing high-frequency *K*-mers in mRNA, species-specific expression characteristics can be captured. In this study, we discovered the presence of HCKs in every single-cell sequencing dataset and further used them as genic sequences for downstream smRNA-seq analysis. By leveraging the strong correlation between marker *K*-mers and *K*-mer-contained motifs, we further explored and obtained reliable results on the functions of the microorganisms.

Compared to well-known tools such as STAR (v 2.7.11b) [3] and Cell Ranger (v 7.1.0), mKmer overcomes the limitations of incomplete or poorly annotated reference genomes by utilizing a cell-by-HCK matrix instead of the traditional cell-by-gene matrix. Currently, MAGs [4] and SAGs [5] can significantly improve the quality of reference genomes. Using high-quality reference genomes allows for the accurate identification of marker genes and their specific functions. Therefore, mKmer still has certain limitations in functional annotation, but its advantage lies in efficiently and unbiasedly identifying and distinguishing species. mKmer requires significantly fewer computational resources and less processing time compared to STAR (v 2.7.11b), making it possible to complete the analysis even on a personal computer (Table 1). Benchmark tests with soybean soil and human gut smRNA-seq data demonstrated that mKmer captures more data than the available tools and achieves clearer species clustering. This is understandable, as both STAR (v 2.7.11b) and Cell Ranger (v 7.1.0) align the obtained smRNA-seq data to the available reference genomes that have been sequenced. This process inevitably introduces biases. Considering the rapid evolution and variation of microorganisms, using single reference genomes per species does not align with established facts; alignment failures due to genetic variations would result in discarding a significant amount of valuable biological information obtained from the smRNA-seq. In contrast, mKmer, which operates without the need for reference genomes, provides a more comprehensive and unbiased analysis of microbiome samples.

It is well known that single-cell RNA sequencing (scRNA-seq) data contain numerous empty droplets and some doublets, as well as other impurity-containing droplets, which can severely impact the quality of sequencing results. Therefore, removing impurity information is crucial for single-cell analysis techniques. By examining the distribution of UMIs and barcodes, high-quality cells can be effectively filtered. Additionally, aligning to a reference genome to create a cell-by-gene matrix is an effective approach to exclude impurities from sequencing results. We know that the longer the *K*-mer, the higher its specificity; thus, longer *K*-mers are more efficient in detecting impurities. Conversely, shorter *K*-mers have higher conservation, which increases information utilization when identifying the same species. mKmer extracts biological information from raw sequencing data by testing different *K*-mer lengths to determine the optimal *K*. This optimal *K* can identify key conserved sequences while effectively distinguishing noise from noncritical gene sequences. As *K*-mer gets longer, the sequencing results from each sample showed a consistent trend (e.g. Fig. 2). Specifically, at a certain *K*, the number of *K*-mers with a frequency of 1 was the highest among all *K*-mer frequencies. We interpret *K*-mers with a frequency of 1 as sequences that are useless for species clustering and may even be impurities. To effectively filter out useless sequences, we ranked each *K*-mer in descending order of frequency and observed a distinct inflection point (Fig. 3A and C). Based on this, we consider the high-frequency *K*-mers before the inflection point as conservative *K*-mers (i.e. HCKs), and cells containing these *K*-mers are likely derived from a common ancestor (i.e. LCA) [52]. Therefore, HCKs exhibit high species recognition. The low-frequency *K*-mers after the inflection point may indicate that the taxonomic information contained in these *K*-mers is sparse, and they may even interfere with species identification and differentiation. It is not recommended to use low-frequency *K*-mers in the downstream dimensionality reduction and clustering process. It is undeniable that there may be errors near the inflection point, where some highly specific conserved *K*-mers may be misclassified as impurities. However, this has minimal impact on species identification across the entire cell set. In our analytical framework, we select these HCKs at inflection points for species identification and functional analysis. At the same time, selection of HCKs addresses issues of computational time and high matrix dimensionality caused by the excessive variety of *K*-mers, significantly enhancing mKmer’s performance and practicality. Additionally, the high frequency of conserved *K*-mers means that they are more likely to come from regions that can translate conserved protein motifs. These protein motifs have been used for cross-species functional annotation of scRNA-seq [53]. We further hypothesize that these HCKs may correspond to motif fragments of certain gene families within microorganisms. Therefore, by functionally annotating these gene motifs, mapping them onto the microbial communities, and performing enrichment analysis, we can infer the specific functions of the species in the sample. The key innovations of mKmer, including HCKs, marker *K*-mers, and *K*-mer-contained motifs, enhance species identification and distinction. This method allows for a holistic view of microbial communities, advancing our understanding of microbial ecology and functional roles. Despite these strengths, mKmer still has room for improvement. For example, species annotation of Kraken 2 [12] can be corrected based on the clustering results of mKmer. Furthermore, several advanced microbial metagenomic analysis tools could be integrated into the downstream analysis of smRNA-seq data in the future. For instance, PathSeq [54] could be employed to filter host-derived reads potentially captured during single-cell sequencing procedures, while high-specificity marker genes of MetaPhlAn3 [55] may enable more precise functional profiling of microbial cells. In future developments, we aim to progressively expand mKmer’s functionality, enhance its analytical accuracy, and optimize computational performance, building upon the aforementioned conceptual framework.

## Conclusion

mKmer is a reference genome-free approach for smRNA-seq analysis and allows studies on cellular heterogeneity, marker motif discovery, and efficiency functional annotation. In this study, we discovered and defined HCK. More accurate clustering results confirmed that HCKs densely encapsulate a large amount of hierarchical information from microbial taxonomy. In benchmark tests on soybean soil and human gut smRNA-seq datasets, mKmer can use more smRNA-seq data than the traditional annotated gene-based methods, achieving more and clearer species clustering for subsequent comprehensive functional analysis. Our method therefore provides an unbiased way to analyze all species in smRNA-seq samples and allows diverse microbiomic single-cell problems to be formulated in a unified way.

**Key Points**

- An unbiased method for single-cell matrix construction: mKmer is a reference genome-free approach for analyzing smRNA-seq data, utilizing *K*-mers for species identification and functional annotation, which overcomes the limitations of traditional tools such as STAR (v 2.7.11b) and Cell Ranger (v 7.1.0), which rely heavily on the microbial reference genome and its annotated genes.
- mKmer can identify high-frequency *K*-mers (HCKs) based on the inherent data characteristics of different samples. By using HCKs, it distinguishes conserved biological sequences from noise, enabling more accurate and efficient species identification at the single-cell level, even in mixtures of species.
- Through benchmarking with human and soil samples, mKmer has been shown to achieve clearer species clustering, capture more data than reference-based methods, and provide a more comprehensive analysis of microbial communities.

## Supplementary Material

Supplementary_Figure_S1_bbaf227

Supplementary_Figure_S2_bbaf227

Supplementary_Figure_S3_bbaf227

Supplementary_Figure_S4_bbaf227

Supplementary_Figure_S5_bbaf227

Supplementary_Figure_S6_bbaf227

Supplementary_Table_S1_bbaf227

Supplementary_Table_S2_bbaf227

Supplementary_Table_S3_bbaf227

Supplementary_file_S1_bbaf227

Supplementary_file_S2_bbaf227

Supplementary_Information_bbaf227

## Data Availability

All smRNA-seq data used by this study are available at NGDC database (https://ngdc.cncb.ac.cn/) under project number PRJCA017256 (accession number SAMC3766839, SAMC3766838, SAMC3766837, SAMC1266599), PRJCAXXX (the soil sample) and GitHub database (https://github.com) under bioinplant/mKmer as demo datasets (soi and gut samples). The mKmer package (v.1.0.0) is available at https://github.com/bioinplant/mKmer. Any additional information required to reanalyze the data reported in this work paper is available from the lead contact upon request.
