# Supplementary figures and images for: mKmer: an unbiased K-mer embedding of microbiomic single-microbe RNA sequencing data
Source: Brief Bioinform. 2025 May 23;26(3):bbaf227. doi: 10.1093/bib/bbaf227 (PMC12100620; doi:10.1093/bib/bbaf227)

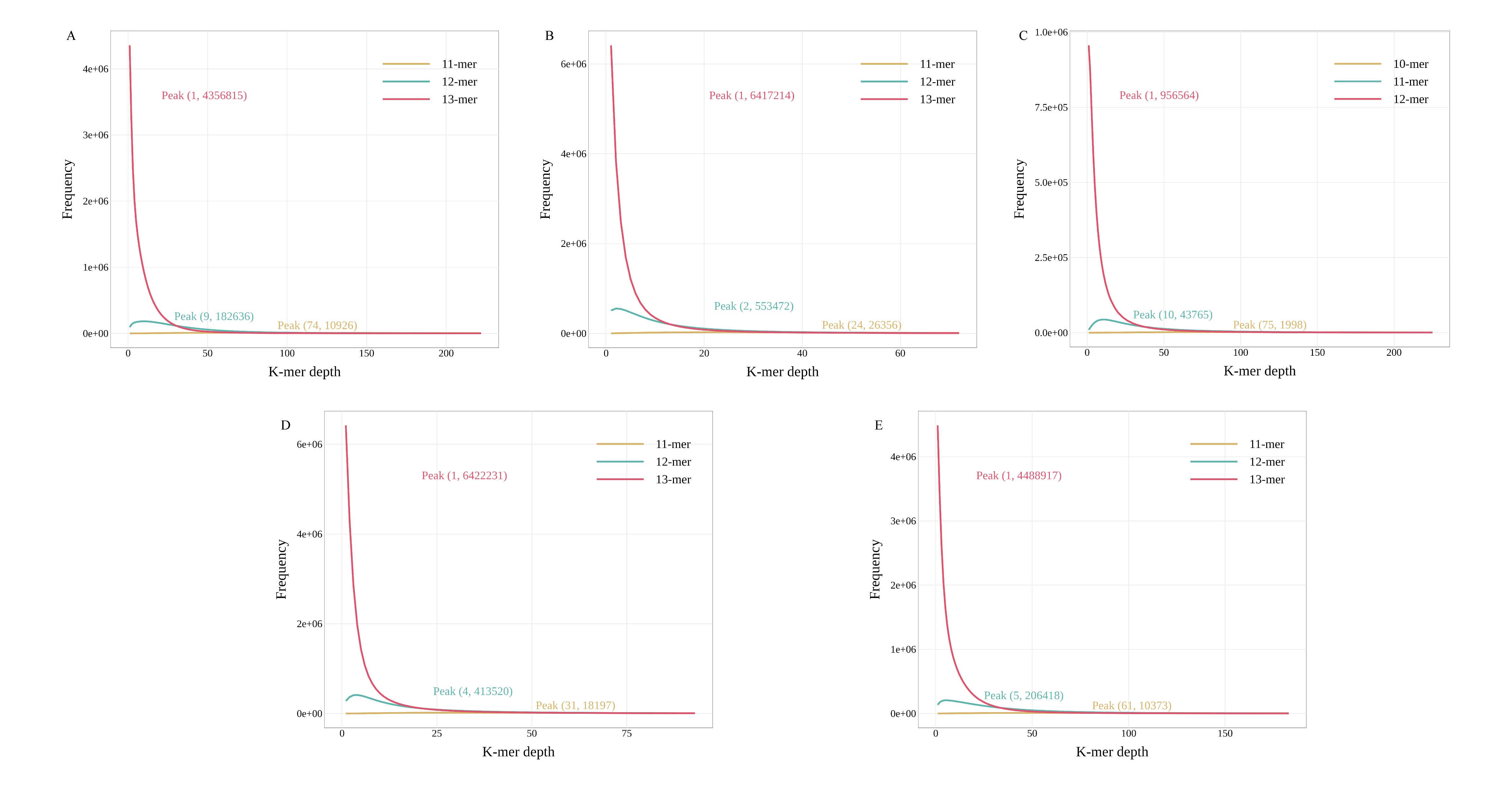

Supplement: Supplementary_Figure_S1_bbaf227 [file supplementary_figure_s1_bbaf227.jpeg]

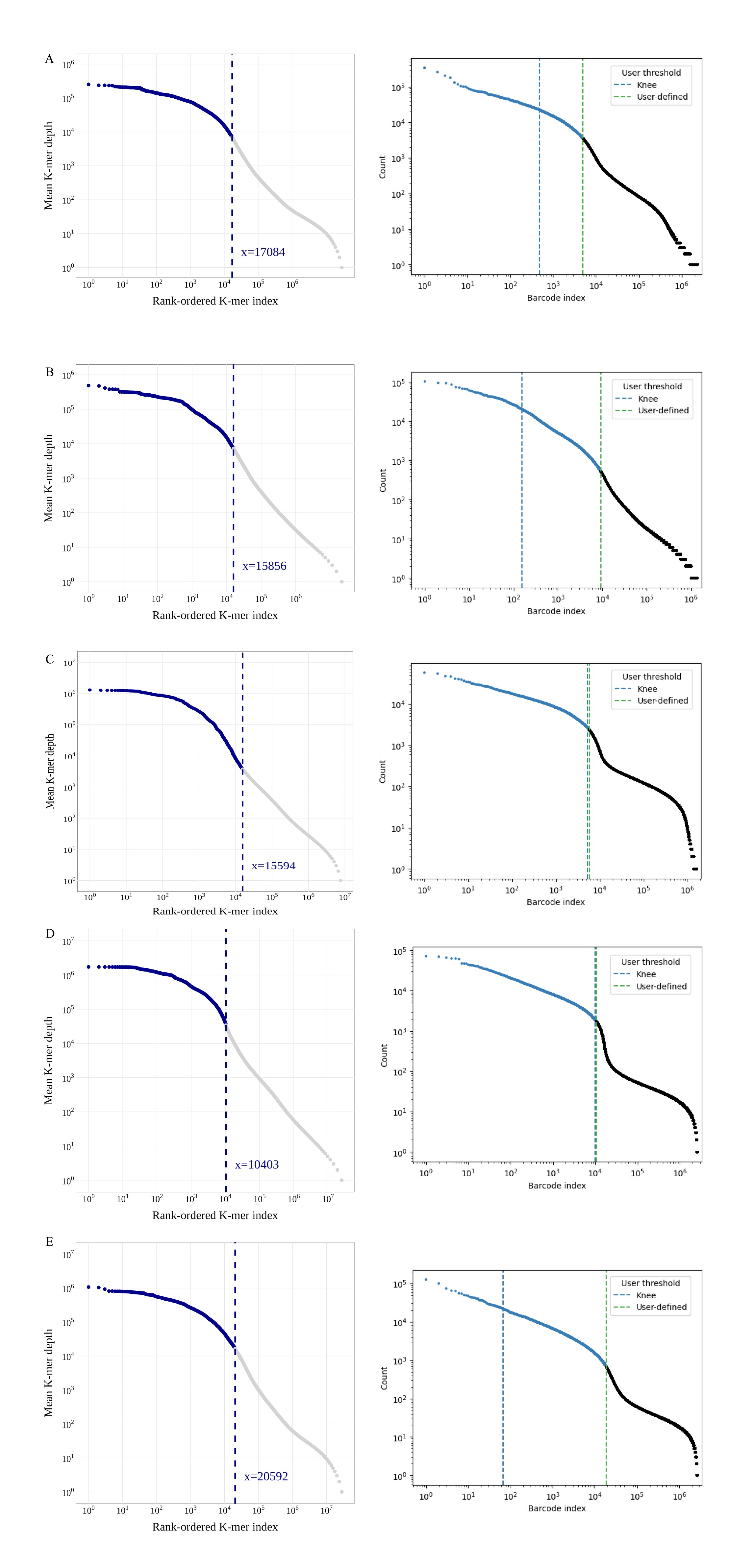

Supplement: Supplementary_Figure_S2_bbaf227 [file supplementary_figure_s2_bbaf227.jpeg]

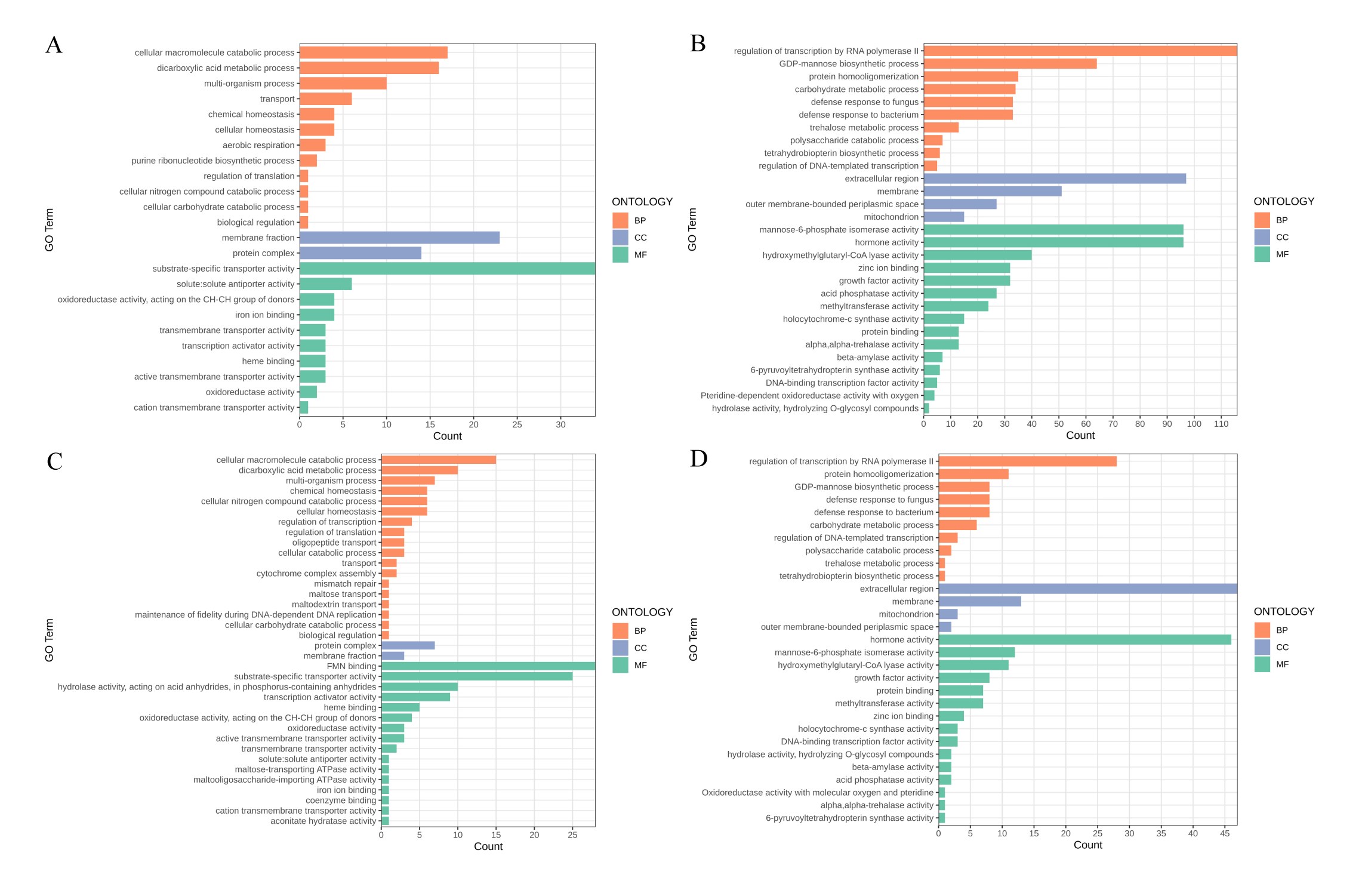

Supplement: Supplementary_Figure_S3_bbaf227 [file supplementary_figure_s3_bbaf227.jpeg]

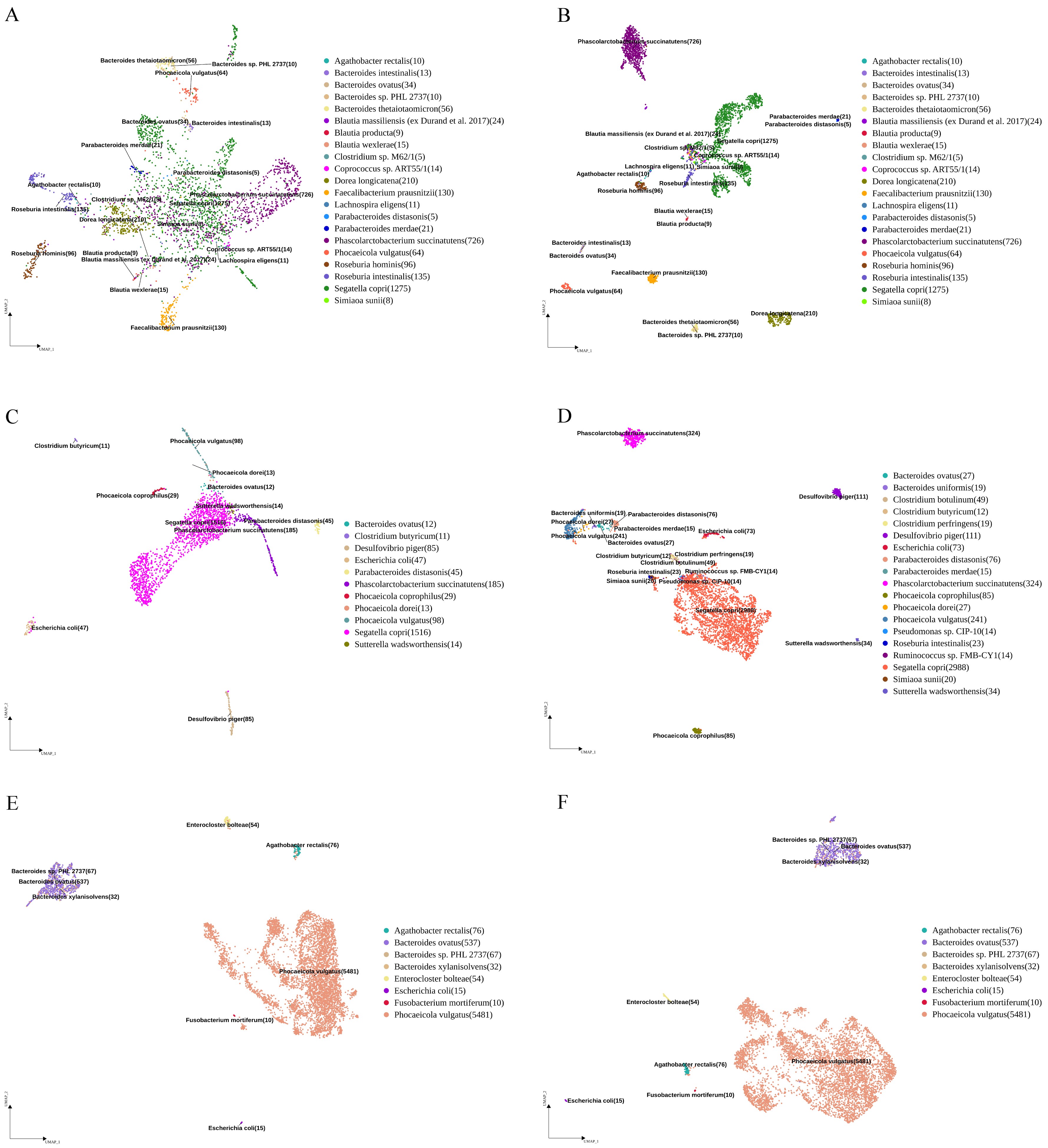

Supplement: Supplementary_Figure_S4_bbaf227 [file supplementary_figure_s4_bbaf227.jpeg]

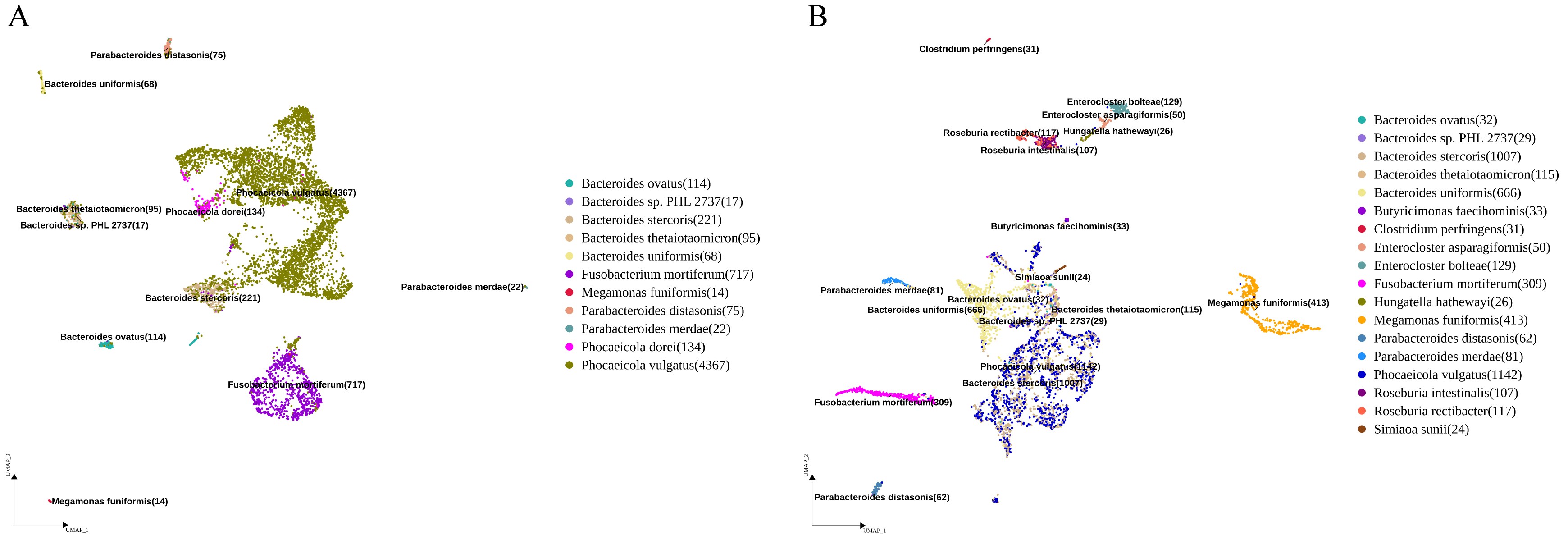

Supplement: Supplementary_Figure_S5_bbaf227 [file supplementary_figure_s5_bbaf227.jpeg]

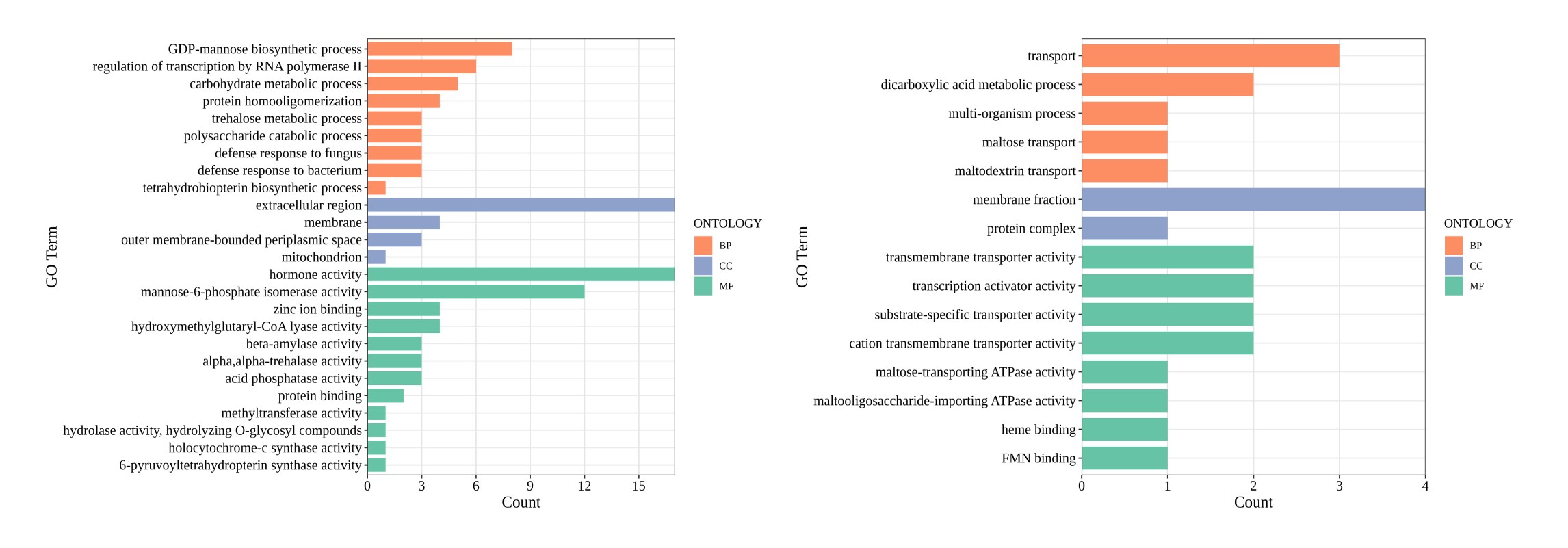

Supplement: Supplementary_Figure_S6_bbaf227 [file supplementary_figure_s6_bbaf227.jpeg]
