# Supplementary material for: mKmer: an unbiased K-mer embedding of microbiomic single-microbe RNA sequencing data
Source: Brief Bioinform. 2025 May 23;26(3):bbaf227. doi: 10.1093/bib/bbaf227 (PMC12100620; doi:10.1093/bib/bbaf227)
Supplement: Supplementary_Table_S1_bbaf227 [file supplementary_table_s1_bbaf227.docx]

**Supplementary Table S1.** Benchmarking of mKmer across biological samples. This table compares the clustering performance of STAR (cell-by-gene matrix) and mKmer (cell-by-HCK matrix) through Davies–Bouldin Index [29] (DBI) and Silhouette Coefficient [30] (SC). Lower DBI or higher SC (bolded) indicate superior cluster compactness and separation.

| **Sample** | **Software** | ***K*-mer length** | **DBI** | **SC** |
| --- | --- | --- | --- | --- |
| Soybean soil | STAR | \ | 10.79 | -0.48 |
|  | mKmer | 13 | 1.15 | 0.39 |
|  |  | 12 | 1.11 | 0.48 |
|  |  | 11 | 0.92 | 0.48 |
| SAMC1266599 | STAR | \ | 3.61 | -0.12 |
|  | mKmer | 13 | 1.71 | 0.34 |
|  |  | 12 | 1.57 | 0.31 |
|  |  | 11 | 1.70 | 0.19 |
| SAMC3766839 | STAR | \ | 2.68 | -0.01 |
|  | mKmer | 12 | 1.42 | 0.21 |
|  |  | 11 | 1.35 | 0.21 |
|  |  | 10 | 1.48 | 0.24 |
| SAMC3766837 | STAR | \ | 2.47 | -0.02 |
|  | mKmer | 13 | 2.26 | 0.03 |
|  |  | 12 | 1.89 | 0.06 |
|  |  | 11 | 2.25 | 0.03 |
| SAMC3766838 | STAR | \ | 2.99 | -0.22 |
|  | mKmer | 12 | 2.32 | 0.10 |
|  |  | 11 | 2.15 | 0.11 |
|  |  | 10 | 2.57 | 0.08 |
| CRC patient  (pre-treatment) | STAR | \ | 1.94 | -0.04 |
|  | mKmer | 13 | 1.78 | 0.17 |
|  |  | 12 | 1.76 | 0.16 |
|  |  | 11 | 1.78 | 0.17 |
| CRC patient  (post-treatment) | STAR | \ | 2.91 | -0.12 |
|  | mKmer | 13 | 1.91 | 0.21 |
|  |  | 12 | 1.77 | 0.25 |
|  |  | 11 | 1.63 | 0.20 |

Note. DBI (The ideal value is 0) and SC (The ideal value is 1) values are rounded to two decimal places.
