# Supplementary material for: mKmer: an unbiased K-mer embedding of microbiomic single-microbe RNA sequencing data
Source: Brief Bioinform. 2025 May 23;26(3):bbaf227. doi: 10.1093/bib/bbaf227 (PMC12100620; doi:10.1093/bib/bbaf227)
Supplement: Supplementary_Table_S2_bbaf227 [file supplementary_table_s2_bbaf227.docx]

**Supplementary Table S2.** Genome coverage of soybean soil msmRNA-seq directly mapped to the reference genome of the target bacterial species

| **Identification status** | **Species** | **Unique mapping (%)** | **Multiple mapping (%)** |
| --- | --- | --- | --- |
| Previously  identified species  (top 3) | *Chitinophaga sp. MD30* | 0.51 | 6.76 |
|  | *Mucilaginibacter rubeus* | 7.72 | 0.32 |
|  | *Pseudomonas aeruginosa* | 0.13 | 10.32 |
| Additionally  identified species  (all 5) | *Bordetella pertussis* | 0.11 | 7.79 |
|  | *Flavobacterium sp. CJ75* | 0.10 | 4.97 |
|  | *Labrys sp. KNU-23* | 0.13 | 13.47 |
|  | *Mucilaginibacter mallensis* | 0.13 | 7.97 |
|  | *Sphingopyxis terrae* | 14.99 | 0.24 |
