# Supplementary material for: mKmer: an unbiased K-mer embedding of microbiomic single-microbe RNA sequencing data
Source: Brief Bioinform. 2025 May 23;26(3):bbaf227. doi: 10.1093/bib/bbaf227 (PMC12100620; doi:10.1093/bib/bbaf227)
Supplement: Supplementary_Table_S3_bbaf227 [file supplementary_table_s3_bbaf227.docx]

**Supplementary Table 3.** Genome coverage of human gut msmRNA-seq directly mapped to the reference genome of the target bacterial species

| **Identification status** | **Species** | **Unique mapping (%)** | **Multiple mapping (%)** |
| --- | --- | --- | --- |
| Previously  identified species  (top 3) | *Phocaeicola vulgatus* | 6.89 | 46.78 |
|  | *Bacteroides stercoris* | 6.08 | 36.49 |
|  | *Parabacteroides merdae* | 0.37 | 22.41 |
| Additionally  identified species  (all 9) | *Roseburia intestinalis* | 0.85 | 15.51 |
|  | *Agathobacter rectalis* | 0.69 | 14.96 |
|  | *Faecalibacterium prausnitzii* | 0.63 | 5.36 |
|  | *Mediterraneibacter gnavus* | 0.61 | 15.01 |
|  | *Phocaeicola coprophilus* | 0.60 | 39.35 |
|  | *Parabacteroides distasonis* | 0.39 | 17.99 |
|  | *Odoribacter splanchnicus* | 0.18 | 12.04 |
|  | *Phascolarctobacterium faecium* | 0.16 | 4.25 |
|  | *Sutterella wadsworthensis* | 0.15 | 2.95 |
