# Supplementary material for: mKmer: an unbiased K-mer embedding of microbiomic single-microbe RNA sequencing data
Source: Brief Bioinform. 2025 May 23;26(3):bbaf227. doi: 10.1093/bib/bbaf227 (PMC12100620; doi:10.1093/bib/bbaf227)
Supplement: Supplementary_Information_bbaf227 [file supplementary_information_bbaf227.docx]

**Supplementary Information**

**Table of Contents**

1. Supplementary Table S1

2. Supplementary Table S2

3. Supplementary Table S3

4. Supplementary Figure S1

5. Supplementary Figure S2

6. Supplementary Figure S3

7. Supplementary Figure S4

8. Supplementary Figure S5

9. Supplementary Figure S6

10. Supplementary File S1

11. Supplementary File S2**Supplementary Table S1.** Benchmarking of mKmer across biological samples. This table compares the clustering performance of STAR (cell-by-gene matrix) and mKmer (cell-by-HCK matrix) through Davies–Bouldin Index (DBI) evaluation. Lower DBI values (bolded) indicate superior cluster compactness and separation.

| **Sample** | **Software** | ***K*-mer length** | **DBI** |
| --- | --- | --- | --- |
| Soybean soil | STAR | \ | 10.79 |
|  | mKmer | 13 | 1.15 |
|  |  | 12 | 1.11 |
|  |  | 11 | 0.92 |
| SAMC1266599 | STAR | \ | 3.61 |
|  | mKmer | 13 | 1.71 |
|  |  | 12 | 1.57 |
|  |  | 11 | 1.70 |
| SAMC3766839 | STAR | \ | 2.68 |
|  | mKmer | 12 | 1.42 |
|  |  | 11 | 1.35 |
|  |  | 10 | 1.48 |
| SAMC3766837 | STAR | \ | 2.47 |
|  | mKmer | 13 | 2.26 |
|  |  | 12 | 1.89 |
|  |  | 11 | 2.25 |
| SAMC3766838 | STAR | \ | 2.99 |
|  | mKmer | 12 | 2.32 |
|  |  | 11 | 2.15 |
|  |  | 10 | 2.57 |
| CRC patient  (pre-treatment) | STAR | \ | 1.94 |
|  | mKmer | 13 | 1.78 |
|  |  | 12 | 1.76 |
|  |  | 11 | 1.78 |
| CRC patient  (post-treatment) | STAR | \ | 2.91 |
|  | mKmer | 13 | 1.91 |
|  |  | 12 | 1.77 |
|  |  | 11 | 1.63 |

**Supplementary Table S2.** Genome coverage of soybean soil msmRNA-seq directly mapped to the reference genome of the target bacterial species

| **Identification status** | **Species** | **Unique mapping (%)** | **Multiple mapping (%)** |
| --- | --- | --- | --- |
| Previously  identified species  (top 3) | *Chitinophaga sp. MD30* | 0.51 | 6.76 |
|  | *Mucilaginibacter rubeus* | 7.72 | 0.32 |
|  | *Pseudomonas aeruginosa* | 0.13 | 10.32 |
| Additionally  identified species  (all 5) | *Bordetella pertussis* | 0.11 | 7.79 |
|  | *Flavobacterium sp. CJ75* | 0.10 | 4.97 |
|  | *Labrys sp. KNU-23* | 0.13 | 13.47 |
|  | *Mucilaginibacter mallensis* | 0.13 | 7.97 |
|  | *Sphingopyxis terrae* | 14.99 | 0.24 |

**Supplementary Table S3.** Genome coverage of human gut msmRNA-seq directly mapped to the reference genome of the target bacterial species

| **Identification status** | **Species** | **Unique mapping (%)** | **Multiple mapping (%)** |
| --- | --- | --- | --- |
| Previously  identified species  (top 3) | *Phocaeicola vulgatus* | 6.89 | 46.78 |
|  | *Bacteroides stercoris* | 6.08 | 36.49 |
|  | *Parabacteroides merdae* | 0.37 | 22.41 |
| Additionally  identified species  (all 9) | *Roseburia intestinalis* | 0.85 | 15.51 |
|  | *Agathobacter rectalis* | 0.69 | 14.96 |
|  | *Faecalibacterium prausnitzii* | 0.63 | 5.36 |
|  | *Mediterraneibacter gnavus* | 0.61 | 15.01 |
|  | *Phocaeicola coprophilus* | 0.60 | 39.35 |
|  | *Parabacteroides distasonis* | 0.39 | 17.99 |
|  | *Odoribacter splanchnicus* | 0.18 | 12.04 |
|  | *Phascolarctobacterium faecium* | 0.16 | 4.25 |
|  | *Sutterella wadsworthensis* | 0.15 | 2.95 |


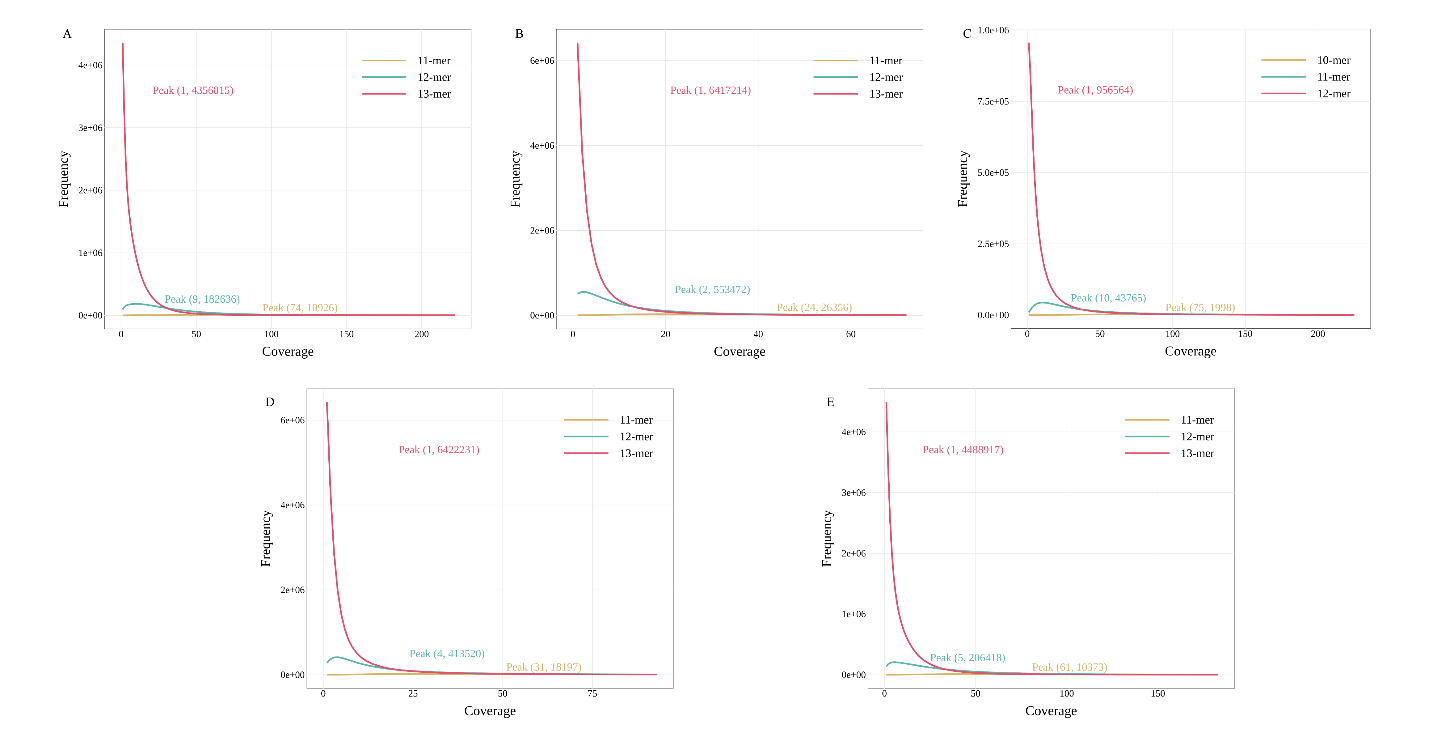


**Supplementary Figure S1.** Frequency of *K*-mers depths by different k sizes for a msmRNA dataset from human gut sample (**A** SAMC1266599; **B** SAMC3766837; **C** SAMC3766838; **D** PRJCAXXX; **E** PRJCAXXX).

**
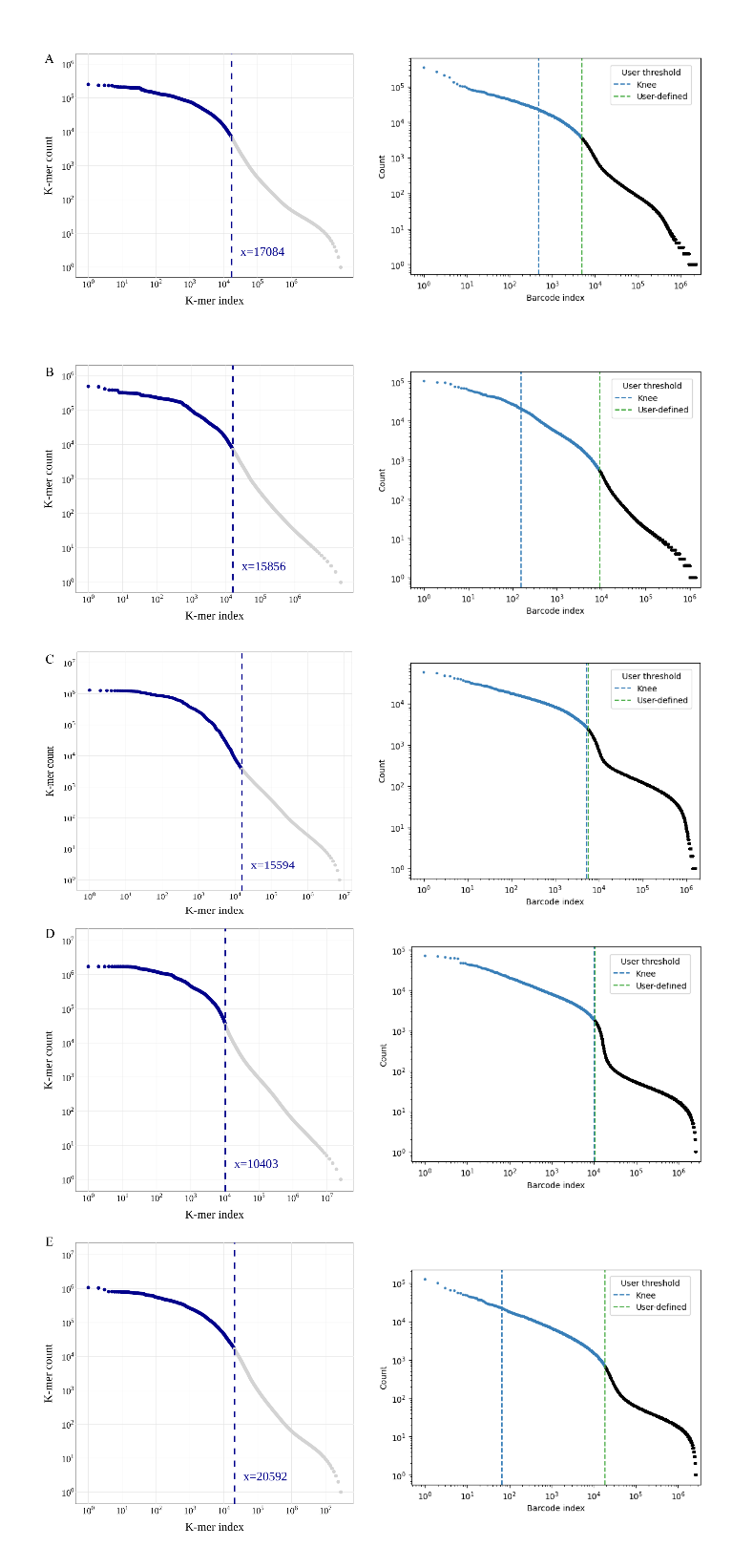
**

**Supplementary Figure S2.** *K*-mer rank plot and barcode rank plot for HCKs calling of the human gut msmNRA sample (**A** SAMC1266599, *K* = 13. **B** SAMC3766837, *K* = 13. **C** SAMC3766838, *K* = 12. **D** PRJCAXXX, *K* = 13. **E** PRJCAXXX, *K* = 13). The *K*-mers in front of the dashed lines are the selected as HCKs.


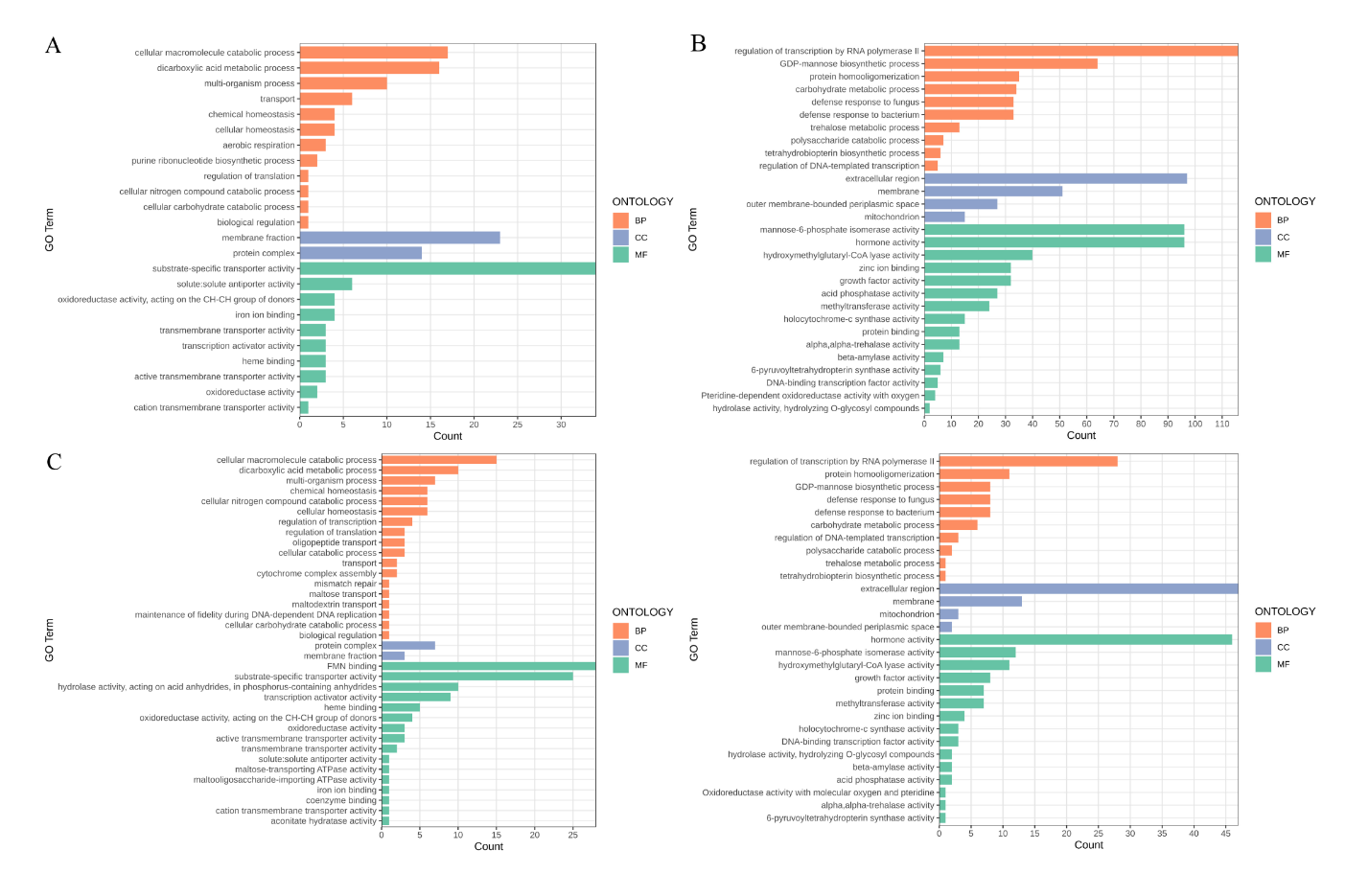


**Supplementary Figure S3.** Functional annotation of soybean soil using *KmerGOn* (A and C) and *KmerGOp* (B and D).


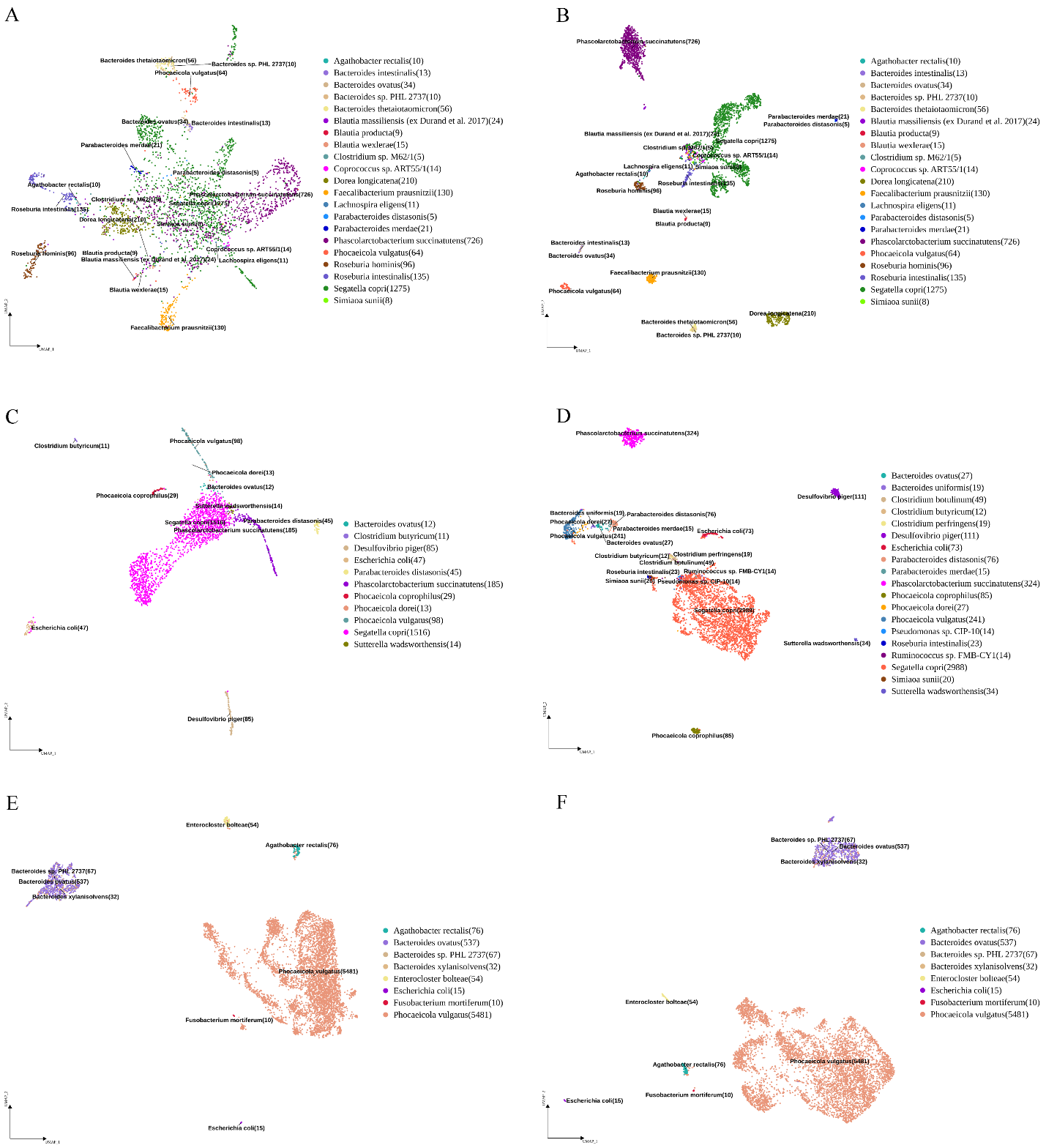


**Supplementary Figure S4.** Benchmark results of three human gut samples by gene-based methods (A, C and E) and mKmer (B, D and F) (**A and B:** SAMC1266599; **C and D:** SAMC3766837; **E and F:** SAMC3766838).


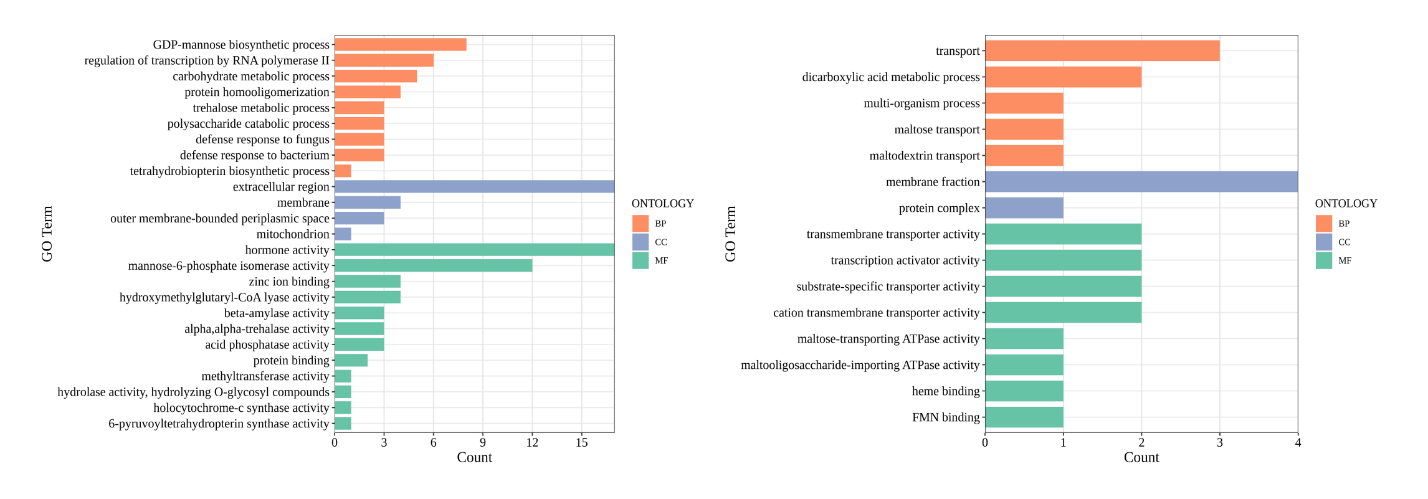


**Supplementary Figure S5.** Functional annotation of human gut (SAMC3766839) using *KmerGOn* (left) and *KmerGOp* (right).


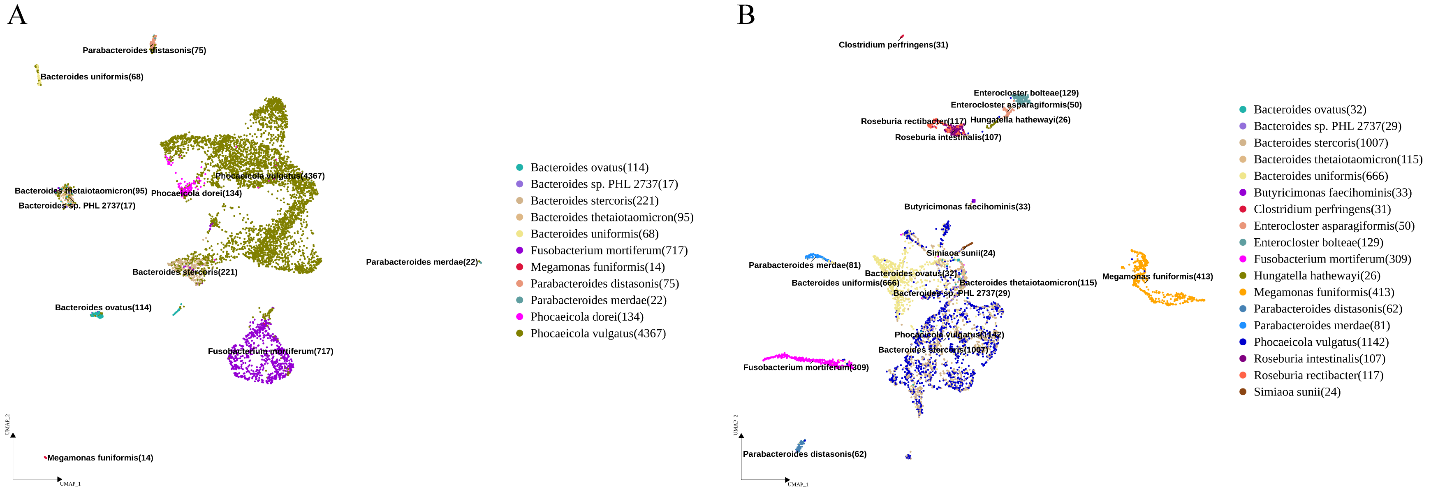


**Supplementary Figure S6.** Human gut samples study by gene/cell matrix. A UMAP clustering and species annotation of the pre-treatment patient gut msmRNA data (PRJCAXXX). B UMAP clustering and species annotation of the post-treatment patient gut msmRNA data (PRJCAXXX).**Supplementary File S1.** Species-specific gene signatures across biological taxa of soybean soil smRNA-seq. This table contains (1) taxonomic identification (Species column), (2) uniquely expressed gene symbols (Gene column), and (3) mean normalized expression levels (Expression column) sorted in descending order per species. Genes were considered species-specific when exclusively expressed (detected in ≥1 cell) in one taxonomic group and completely absent (0 expression) in all others. Data generated through single-cell RNA-seq analysis using Seurat v4 (log-normalized counts).

**Supplementary File S2.** Species-specific gene signatures across biological taxa of human gut smRNA-seq. The table information is the same as Supplementary File 1.
